# Disrupting Treg lineage stability elicits RORgT-mediated plasticity and enhances anti-tumor immunity

**DOI:** 10.1038/s41419-026-09025-0

**Published:** 2026-07-06

**Authors:** Courtney A. Swain, Dominique C. Hinshaw, Ian D. Miranda, Brandon J. Metge, Amr Elhamamsy, Dongquan Chen, Alyssa Lee, Hua Guo, Bhavyasree Papineni, Li’an Williams, Jeffrey C. Rathmell, Selvarangan Ponnazhagan, Robert S. Welner, Rajeev S. Samant, Lalita A. Shevde

**Affiliations:** 1 Medical Scientist Training Program, University of Alabama at Birmingham, Birmingham, AL USA; 2 Department of Pathology, University of Alabama at Birmingham, Birmingham, AL USA; 3 Department of Medicine, Division of General Internal Medicine and Population Science, University of Alabama at Birmingham, Birmingham, AL USA; 4 O’Neal Comprehensive Cancer Center, University of Alabama at Birmingham, Birmingham, AL USA; 5 Department of Pathology, Microbiology, and Immunology, Vanderbilt University, Nashville, TN USA; 6 Vanderbilt Center for Immunology, Vanderbilt University, Nashville, TN USA; 7 Department of Medicine, Division of Hematology and Oncology, University of Alabama at Birmingham, Birmingham, AL USA; 8 Birmingham Veteran Affairs, Birmingham, AL USA

**Keywords:** Breast cancer, Immune evasion

## Abstract

The imbalance of pro-inflammatory and immunosuppressive constituents in the tumor microenvironment (TME) significantly dictates cancer progression, immune invasion, and treatment response. Immunosuppressive regulatory T cells (Tregs) are associated with decreased disease-free survival due to their ability to suppress anti-tumor immunity. Notably, in response to environmental cues, Tregs display functional and phenotypic plasticity with inflammatory helper T cell subsets. Given their predominant role in sustaining immune suppression, it is remarkable that multiple developmental pathways converge to regulate Treg development and function. In this study, we designed, validated, and employed a novel genetically modified mouse model to conditionally ablate the Hedgehog (Hh) gene, Gli2, specifically in Tregs. Ablation of Gli2 activity in Tregs significantly reduced tumor burden, impaired Treg suppressive function, and shifted the transcriptional balance of Foxo3 and Rorγt, transcription factors essential for Tregs and Th17 cells. Spatial mapping highlighted that Gli2 ablation in Tregs enhances the immunogenicity of the tumor and promotes a pro-inflammatory milieu of the TME. This was underscored by a higher tumor immune signature score and enhanced infiltration of cytotoxic CD8^+^ T cells into the tumor. These findings highlight Hh/Gli2 signaling in Tregs as a mechanistic regulator of immunogenicity in the TME and a potential therapeutic target to prime tumors for enhanced responsiveness for adjuvant treatments.

## Introduction

Aberrant reactivation of developmental pathways enables tumor cells to grow, invade, assume stem-like properties, and evolve. Much like an immune-tolerant microenvironment that marks embryonic development, the tumor microenvironment (TME) is also dominated by a sustained immune suppressive habitat delicately orchestrated by developmental signaling pathways [1].

Making up nearly 20% of all breast cancer cases, triple-negative breast cancer (TNBC) sustains an immunologically cold microenvironment due to the limited infiltration of cytotoxic lymphocytes primarily attributed to the copious presence of immunosuppressive constituents such as tumor-promoting M2-like macrophages, cancer stem cells, myeloid-derived suppressor cells, and regulatory T cells (Tregs) [1–4]. Tregs are associated with decreased disease-free survival, increased tumor burden, and increased risk of recurrence in breast and other cancers [5, 6]. Tregs, an immunosuppressive subset of CD4^+^ T lymphocytes, play a vital role in modulating autoimmunity due to their suppressive functions such as secretion of granzyme, expression of cytotoxic T-lymphocyte associated protein 4 (CTLA4), and sequestration of the pro-inflammatory cytokine IL-2 via overexpression of the IL-2 receptor, CD25 [5, 7]. Their differentiation and function are transcriptionally regulated by forkhead box protein P3 (FOXP3), the master regulator of Treg identity and stability.

Tregs are acutely sensitive to their milieu and can transdifferentiate into inflammatory T helper (Th) subsets, such as helper T cell type 17 (Th17) cells, helper T cell type 1 (Th1) cells, or the tolerant helper T cell type 2 (Th2) cells [8–16]. Environmental cues such as IFNγ and IL-27 can induce Tregs to acquire Th1-like characteristics by promoting CXCR3 and TBET [12]. In response to other inflammatory microenvironmental stimuli such as IL-6 and TNF-α, Tregs can trans-differentiate to Th17 cells with a composite stage of Th17-like Tregs, or Tr17 cells, that maintain FOXP3 expression while expressing classical Th17 markers such as retinoic acid-related orphan receptor γt (Rorγt) and interleukin-17a (IL-17a) [12, 14, 17–20]. Due to this unique expression profile, Tr17 cells can adopt a more inflammatory rather than suppressive functional program [17–22].

Given their predominant role in sustaining immune suppression, it is quite notable that multiple developmental pathways, including WNT, TGF-β, Notch, and Hedgehog (Hh), converge to regulate Treg development and function. Activation of WNT in Tregs leads to the downregulation of FOXP3, TGF-β, and IL-10. TCF-1, a downstream effector of WNT signaling, inhibits FOXP3 transcriptional activity, thereby mitigating their immunosuppressive function [19]. In induced Tregs, WNT activation supports GATA3 expression and pivots Tregs to differentiate into Th2 cells [20]. Notch signaling inhibits the development and suppressive function of Tregs. Specifically, Notch 4-expressing Tregs upregulate the expression of the Hippo transcription factor TAZ, which functions as a negative regulator of FOXP3 activity [21].

Hh signaling is important in embryonic development in a highly conserved manner across various vertebrates and invertebrates. In the presence of a Hh ligand, the transmembrane receptor Patched (PTCH) is inhibited, relieving the inhibition of the downstream activator and transmembrane protein Smoothened (SMO), which relocates to the primary cilium, where it activates glioma-associated oncogenes (GLI) transcription factors that translocate to the nucleus to induce the transcription of target gene expression [23, 24]. Besides cancer, activation of Hh has been reported in chronic pulmonary fibrosis, allergic lung disease, inflammatory gut disease, and wound healing, highlighting its impact on immune regulation [25–32]. Aberrantly activated Hh signaling is a prominent feature of many cancer types due to genetic aberrations or autocrine or paracrine signaling reflecting a crosstalk with tumor-associated cellular constituents such as cancer-associated fibroblasts [3]. Emerging literature suggests that abundant levels of Hh ligands within the breast TME promote an immunologically cold environment [3, 4]. Inhibiting Hh activity yielded a TME that was richly populated with anti-tumor macrophages and cytotoxic T cells, with a notable decrease in Tregs [22]. Additionally, the activation of Hh signaling suppresses CD8^+^ T cell recruitment in breast and other cancer models [2, 22].

In atopic dermatitis, the Sonic hedgehog (SHH) ligand has a protective effect against the induction of skin inflammation, as the pharmacologic blockade of Hh activity and the conditional repression of Gli2 transcription in T cells exacerbated disease progression [32]. Gli2 has been identified as a driver of Treg differentiation and immunoregulatory functions, indicating that the role of Hh signaling is essential for Treg stability and suppressive potential [2, 32].

In this study, we designed, validated, and employed a novel genetically engineered mouse model (GEMM) to conditionally ablate Gli2 specifically in Tregs. Gli2 deletion orchestrated molecular reprogramming in Tregs, significantly impairing their immunosuppressive function and promoting trans-differentiation into inflammatory Tr17 cells. This was accompanied by an immunostimulatory and inflammatory TME, with a significant increase in proliferative and cytotoxic CD8^+^ T cells and an anti-tumorigenic cytokine profile. Utilizing spatial transcriptomic analyzes, we characterized changes in the TME induced by this impairment of Tregs. In Gli2-ablated conditions, spatial analyzes identified immune-enriched zones characterized by a pro-inflammatory TME with distinctive transcriptomes and spatial relationships that suggest that Gli2 ablation in Tregs enhances the immunogenicity of the tumor.

## Materials and methods

### Cell culture

Dulbecco’s modified minimum essential medium (DMEM; Life Technologies) supplemented with 10% fetal bovine serum (FBS; Life Technologies) and 100 μg/mL of G418 (Life Technologies) was used to culture 4T1 luciferase-expressing mammary carcinoma cells. DMEM supplemented with 10% FBS was used to culture EMT6 luciferase-expressing murine mammary carcinoma cells. For EL4 cells, DMEM supplemented with 10% horse serum was used. Frozen stocks of cells from early passages were routinely used to replace cultures with increased passage number. All cell lines were tested annually for contamination by *Mycoplasma*.

### Animal usage

Balb/c and/or C57BL/6 mice were used for all animal experiments with approval from and in accordance with the Institutional Animal Care and Use Committee (IACUC) at the University of Alabama at Birmingham (UAB). All animals were humanely euthanized at the endpoints for the respective experiments.

### Generation of the Treg-specific Gli2 mouse model

Swiss Webster mice containing the locus of crossing over P (loxP) sites upstream of exon 7 and downstream of exon 8 on the allele for Gli2 (*Gli2*^*fl/fl*^) were generated and provided by Dr. Alexandra Joyner from the Memorial Sloan-Kettering Cancer Center. To ablate Gli2 in Treg cells, a GEMM was designed to harbor a CreERT2 construct to regulate *Foxp3*. For this, the Swiss Webster Gli2^f/f^ mice were crossed with Foxp3^tm9(EGFP/Cre/ERT2)AYR^ mice on a C57BL/6 background (Strain #016961; Jackson Laboratory). After confirming the Gli2^f/f^ Foxp3-Cre^+^ (Foxp3-*Gli2*^*fl/fl*^) genotype on the mixed Swiss Webster-C57BL/6 background, the mice were transferred to a pure Balb/c background. Tail clippings from the pups of these crossings were sent to Jackson Laboratory for genome scanning via speed congenics to identify mice with the highest percentage of Balb/c background, which were then used for further breeding and colony generation. The genotype was confirmed in all experimental mice, including the control group, by PCR before initiating studies. All mice were at least 6 weeks of age at the start of the study or when euthanized for CD4^+^ T cell isolation.

### CD4^+^ T cell isolation, Treg differentiation, and treatments

For each relevant experiment, spleens were harvested from euthanized mice and dissociated using a 70 μm strainer (Falcon, Durham, North Carolina). Red blood cells were lysed using ACK buffer (Life Technologies). Enrichment of naïve CD4^+^ T cells (Th0) was performed using the Naïve CD4^+^ T cell Isolation Mouse Magnetic Kit (Miltenyi Biotec, Gaithersburg, Maryland). To differentiate Th0 cells into inducible regulatory T cells (iTregs), 5 × 10^5^ cells/mL of naïve CD4^+^ T cells were seeded into αCD3/CD28 (5 μg/mL, Biolegend, San Diego, California) coated 6-well plates using TexMACS^TM^ media (Miltenyi Biotec) with 10% FBS, 1% penicillin and streptomycin, 2 mM GlutaMAX^TM^ (Thermo Fisher), and 50 μM 2-mercaptoethanol (Sigma-Aldrich, St. Louis, Missouri). Th0 cells were placed in Treg-polarizing conditions for 72 h where complete TexMACS^TM^ media was supplemented with 20 ng/mL of recombinant mouse IL-2 (rmIL-2; R&D Systems, Minneapolis, Minnesota), 6 ng/mL of recombinant human TGF-β (rhTGF-β; R&D Systems), 10 μg/mL of anti-IL-4 (Biolegend), and 10 μg/mL of anti-IFNγ. Following differentiation, iTregs were treated for 24–48 h with either 2 μM 4-hydroxytamoxifen (*Gli2*^−/−^ iTregs; 4-OHT, Sigma-Aldrich) or 100% ethanol (*Gli2*^*fl/fl*^ iTregs) as a control.

### In vivo model validation

Foxp3-*Gli2*^*fl/fl*^ mice were administered tamoxifen (TAM; Sigma-Aldrich) via intraperitoneal injection to induce ablation of Gli2 in Tregs. The regimen involves the treatment of mice on days 0, 1, 3, and 17 with 6 mg of TAM dissolved in corn oil (Sigma-Aldrich) with 5% ethanol. Control Foxp3-*Gli2*^*fl/fl*^ mice were treated with the vehicle control (corn oil with 5% ethanol). On day 18, Tregs from the spleen were enriched using the EasySep Mouse CD4^+^ CD25^+^ Regulatory T cell Isolation Kit II (Stem Cell Technologies, Kent, WA) and subjected to downstream analyzes.

### In vivo tumor studies

For in vivo studies to assess Treg activity, Foxp3-*Gli2*^*fl/fl*^ mice were injected with 5 × 10^5^ 4T1 or 2 × 10^5^ EMT6 cells into the inguinal mammary fat pad. Tumors were monitored and measured throughout the course of the experiment. Mean tumor volume (MTV) was assessed using caliper measurements. Before initiating treatment, mice were randomized to the control or TAM treatment group based on MTV on day 6 to ensure a comparable MTV between groups. Mice were treated with 6 mg TAM on days 6, 7, and 9, and then with 3 mg TAM on days 10, 12, 14, and 16 until termination. Spleens and tumors were dissected, weighed, and dissociated on day 17. Tumors were dissociated using the Mouse Tumor Dissociation Kit (Miltenyi Biotec) and the gentleMACS Octo Dissociator with Heaters (Miltenyi Biotec). Splenocytes and dissociated tumor cells were assessed via flow cytometry as detailed in the ‘Flow cytometry’ section below.

### Treg suppression assay

Tumors and spleens from 4T1 or EMT6 tumor-bearing Foxp3-*Gli2*^*fl/fl*^ or Foxp3-*Gli2*^*−/−*^ mice were dissected and dissociated. CD4^+^ CD25^−^ effector T cells (T_eff_) were isolated from non-tumor-bearing, non-treated mice. Tumor- or spleen-derived Tregs were cocultured with T_eff_ labeled with 5 μM carboxyfluorescein (CFSE; Biolegend) according to the manufacturer’s protocol for 96 h and assessed by flow cytometry.

### Western blotting

Cells were lysed in 1X RIPA lysis buffer (Millipore Sigma) containing 1X Halt Protease Inhibitor Cocktail (Thermo Fisher, Rockford, IL). The lysates were immunoblotted for Gli2, Foxp3, TGFβ, and the loading control β-actin (details for all antibodies in Supplementary Table 1). Primary antibodies were diluted 1:1000, and the ECL^TM^ anti-mouse IgG secondary antibody (Cytiva, Marlborough, MA) was diluted 1:5000. The blots were developed using GE Amersham ECL prime western blotting detection reagent (Cytiva, Buckinghamshire, United Kingdom) and imaged using the Amersham Imager 600 (GE Health Care, Uppsala, Sweden).

### Quantitative real-time polymerase chain reaction (qRT-PCR)

RNA was isolated using the RNeasy Mini Kit (Qiagen, Germantown, Maryland) and quantified using the NanoDrop (Thermo Fisher). RNA was reverse transcribed into cDNA using the High-Capacity cDNA Reverse Transcription Kit (Life Technologies) using the following protocol: 25 °C for 10 min, 37 °C for 120 min, and 85 °C for 5 min. Quantitative PCR was performed using either a SYBR Green Assay (Thermo Scientific; for *Gli2*) or TaqMan Real-Time PCR Assays (Thermo Scientific; for *Foxp3*, *Il2ra, Ebi3, Tgfb1, Foxo1, Foxo3, Rorc*, and *Il17a*)*. Actb* was used as a control for all assays. Steady-state expression is depicted as 2^-ΔΔct^.

### Flow cytometry

After counting and assessing viability, cells were plated into a 96-well U-bottom plate (Falcon) and stained with the eFluor 450 viability dye (Thermo Fisher) in 1X PBS for 15 min on ice in the dark. Cells were then washed and stained for extracellular markers with the primary conjugated antibody cocktail (BioLegend) for 30 min. The cocktail was dissolved in FACS buffer (1X PBS with 5% BSA and 1% sodium azide) or in Brilliant Violet Buffer (BioLegend) if Brilliant Violet dyes were being used. Cells were then fixed with nuclear fixation buffer using the True-Nuclear Transcription Factor Buffer Set (BioLegend) for at least 45 min at room temperature in the dark, followed by washing with True-Nuclear permeabilization buffer (BioLegend). A primary antibody cocktail in nuclear permeabilization buffer was used to stain the cells for intracellular and nuclear markers for 30 min on ice in the dark. After this final antibody staining step and washing, the cells were resuspended in FACS buffer. Unstained controls and fluorescence minus one controls were used for all flow cytometry steps. Data was acquired using the Attune NxT Flow Cytometer (Invitrogen, Carlsbad, CA) and analyzed using FlowJo version 10.8 (BD Biosciences, Ashland, OR). The antibodies used are listed in Supplementary Table 1.

For assessing intracellular cytokine production, cells were stimulated for 5 h at 37 °C with the Cell Activation Cocktail with Brefeldin A (BioLegend) according to the manufacturer’s protocol before proceeding to the flow cytometry staining protocol.

#### Gating strategies

Pan-CD3^+^ T cells: Lymphocytes based on size > Single cells > Live > CD3^+^

CD4^+^ T cells: Lymphocytes based on size > Single cells > Live > CD4^+^

Treg cells: Lymphocytes based on size > Single cells > Live > CD4^+^ > Foxp3^+^

Th17 cells: Lymphocytes based on size > Single cells >Live > CD4^+^ > Rorγt^+^, IL-17a^+^

Tr17 cells: Lymphocytes based on size > Single cells > Live > CD4^+^ > Foxp3^+^ > Rorγt^+^, IL17a^+^

CD8^+^ T cells: Lymphocytes based on size > Single cells > Live > CD8^+^

Ki-67, IFNγ, TIM3, and Granzyme B (GZMB) were used as functional markers for proliferation, inflammatory activity, exhaustion, and cytotoxicity, respectively.

### Cytokine array

Tumors were harvested, dissociated, and lysed for protein in 1X PBS with Halt Protease Inhibitors and 1% Triton X-100 (Sigma-Aldrich). The Mouse XL Proteome Profiler Cytokine Array (R&D Systems) was used to assay 200 μg of protein per tumor. The array was imaged using the Amersham Imager 600 chemiluminescence system, and ImageJ was used to quantify densitometry.

### Bulk RNA-sequencing

Splenic Th0 cells were enriched from the spleens of non-tumor-bearing, non-treated Foxp3-*Gli2*^*fl/fl*^ mice and induced into iTregs. After treating with 4-OHT (or vehicle control), RNA was extracted using the RNeasy Mini Kit (Qiagen). Library preparation and standard RNA-sequencing with poly(A) selection and 150-bp paired-end reads were performed on an Illumina HiSeq 2500 by Azenta Life Sciences (South Plainfield, NJ). All raw sequences were processed by Trimmomatic [33], aligned and sorted by using the HISAT2 [34] and Samtools [35]. Generated counts matrix and phenotypic data were imported into RStudio for differential gene expression analysis using the R package *DESeq2* [36]. Output from *DESeq2* differential gene expression analysis and gene set enrichment analysis (GSEA) results are detailed in Supplementary Table 2.

### Single-cell RNA-sequencing

4T1 tumors were subjected to single-cell RNA-Sequencing (scRNA-Seq) as previously described [22]. The 10X Genomics Cell Ranger pipeline *mkfastq* (version 4.0.0) was used to create the fastq files from the sequencer. Cell Ranger *count* pipeline (version 7.1.0) was used to align the raw sequence reads to the Mus musculus (mm10) reference genome using STAR.

### Spatial transcriptomics

Formalin fixed 4T1 tumors from Foxp3-*Gli2*^*−/−*^ or Foxp3-*Gli2*^*fl/fl*^ mice (from the Treg activity treatment regimen) were preserved in 70% ethanol until sectioning. Before processing according to the manufacturer’s protocol, 10 μm sections of two 4T1 tumors from Foxp3-*Gli2*^*−/−*^ and two Foxp3-*Gli2*^*fl/fl*^ were placed onto 10X Genomics Spatial Gene Expression slides. Slides were stained with hematoxylin and eosin (H&E), permeabilized, and second-strand DNA was generated from cDNA. Board-certified pathologists performed a double-blinded histological review of each tumor section. Using annotated images provided by the pathologists, manual alignment of the capture area for each tumor section was applied to exclude irrelevant non-tumor tissue (i.e., muscular and lymphoid tissue) and debris in the downstream transcriptomic analysis. First, the 10X Genomics Space Ranger *mkfastq* pipeline was used to generate fastq files from the BCL files. Reads were processed through the 10X Genomics Space Ranger *count* pipeline (v3.1), which performed alignment to the mouse reference genome (mm10), unique molecular identifier (UMI) counting, and fiducial detection.

### Analysis of scRNA-Seq and spatial transcriptomics

Spatial and scRNA-Seq data were analyzed using RStudio v4.4.2 (Boston, MA, USA) and the transcriptomic analytical R package *Seurat* v5.0.1. The spatial and scRNA-Seq data were imported into Seurat in the HDF5 feature-barcode matrix format and loaded into RStudio using the *Read10X_h5* function. The *CreateSeuratObject* function was used to generate Seurat objects for each tumor sample. Each Seurat object was merged using the *merge* function and integrated using the R package *harmony*. Cells were filtered based on feature counts and mitochondrial percentage, where cells with more than 4000 features or less than 200 features were excluded from the analysis. Additionally, the datasets were scaled and normalized. Principal component analysis (PCA) of the variable genes as input was performed based on 15 principal components and visualized by graph-based clustering and UMAP dimensional reduction.

For spatial transcriptomics analyzes, two tumors from Foxp3-*Gli2*^*fl/fl*^ and Foxp3-*Gli2*^*−/−*^ mice were sectioned, stained by H & E, and imaged before proceeding to library prep and sequencing. For each tissue slide, manual histological annotations were completed by board-certified pathologists that accurately denoted the manual alignment for each capture area with a focus on the tumor tissue. All four tumors were spatially barcoded and subjected to analysis using Seurat v5. A list of top DEGs between clusters generated by Seurat was used to annotate the resultant clusters utilizing literature review and PanglaoDB. Given the resolution of the Visium V1 technology, transcriptional signatures were used to annotate the enrichment of more prominent cell populations, such as tumor cells, macrophages, and lymphocytes. Clusters were first characterized based on immune- or epithelial-specific biomarkers (see Supplementary Table 2). Top DEGs observed in each cluster revealed tumor, macrophage, and immune cell-relevant genes. These gene lists were used to annotate and deconvolute each cluster. Applying tumor- (*Epcam, Krt7, Krt8, Krt14*), macrophage- (*Adgre1, Mrc1, Cd163, Il4ra, Stat1*), B cell- (*Cd19, Cd20*), and T cell-focused (*Cd3e, Cd4, Cd8a, Foxp3*) signatures to the dataset led to the identification of four zones with unique composition of these prominent cell populations: “Immune-enriched”, “Tumor_Macrophage-enriched”, “Tumor-enriched”, and “Tumor_Immune-enriched”. The WebGestalt platform was used to perform gene ontology (GO) analyzes on each zone to identify biological processes involved in each region based on their transcriptomic profile. GSEA, Kyoto Encyclopedia of Genes and Genomes (KEGG) pathway database, and Gene Ontology (GO) term annotations were performed using the WebGestalt web portal for all datasets [37]. Visualizations were generated using *ggplot2*, *ggVenn*, *EnhancedVolcano*, or *Seurat*. Output from DEG analyzes and GSEA results are detailed in Supplementary Tables 2–4.

### Publicly available transcriptomic datasets

To determine correlations between Tregs, *GLI2* expression, and various markers of immunomodulation, an integrated repository portal for tumor-immune system interactions (TISIDB) [33] was used. To query potential binding sites of Gli2 on *Foxo1*, the binding sequence was found using the JASPAR database to identify potential binding sites within a CHIP-Sequencing dataset of human THP-1 cells (GSM2212253) [38].

### Chromatin immunoprecipitation and quantitative PCR (ChIP-qPCR)

Chromatin immunoprecipitation and quantitative PCR (ChIP-qPCR) was used to determine the binding of transcription factors to the regulatory region of their target genes. For iTreg studies, Th0 cells from the spleens of wildtype mice were differentiated into iTregs and treated with 20 μM GANT61 (Hh-i, Sigma Aldrich), 20 μM Vismodegib (Smo-i), or vehicle control (DMSO) for 24 h. For Foxp3-*Gli2*^*−/−*^ iTreg studies, Th0 cells from the spleens of Foxp3-*Gli2*^*fl/fl*^ mice were differentiated into iTregs and treated with 4-OHT or vehicle control as described above. To assess differential binding of Rorγt on the *Il17a* promoter in a Th17 model system, EL4 cells were differentiated into Th17 cells by plating cells in DMEM media supplemented with 10% FBS and into 5 μg/mL anti-CD3/CD28 coated plates for 72 h. To inhibit Hh signaling, Th17 polarized EL4 cells were plated with 20 μM Hh-i, 20 μM Smo-i, or vehicle control (DMSO) for 24 h. After treatment, all cells were harvested and used for ChIP-qPCR analysis.

ChIP was performed using the SimpleChIP® Plus Enzymatic Chromatin IP Kit with magnetic beads (Cell Signaling) following the manufacturer’s protocol. Briefly, cells were resuspended in serum-free TexMACS media, and proteins were crosslinked to DNA by adding 37% formaldehyde to the suspension for a final 1% formaldehyde concentration. To stop the reaction, 10X glycine was added, and the samples were washed with PBS. For Th17 polarized EL4 cells, 1 µL of Micrococcal nuclease was added per sample and incubated in a 37 °C water bath for 20 min. Each sample was then sonicated six times using a Model 120 Sonic Dismembrator (Fisherbrand) for 10 s at 40% amplitude with 60 s rest on ice between each sonication. Proper chromatin digestion was ensured by running samples on a 1% agarose gel. ChIP was performed per the manufacturer’s protocol using 5 μg of Rorγt monoclonal antibody (mAb; ThermoFisher, Waltham, MA) or IgG isotype control (Rat IgG2a, ThermoFisher, Waltham, MA). For iTregs, 0.25 μL of micrococcal nuclease was added per IP and incubated in a 37 °C water bath for 10 min. Each sample was sonicated four times for 10 s at 40% amplitude with 60 s rest on ice between each sonication. ChIP was performed per the manufacturer’s protocol using either 3 µg of Foxo3 (Proteintech Group Inc., Rosemont, IL), 5 μg of Rorγt mAb, or IgG2a isotype control.

Chromatin was eluted from the immunoprecipitation, crosslinking was reversed, and the resultant DNA was purified using the Qiagen DNA purification kit (Qiagen). Purified DNA from ChIP and input was subjected to RT-qPCR using 2X Maxima SYBR Green Master Mix (ThermoFisher) with primer pairs to amplify Rorγt or Foxo3 DNA binding regions on *Il17a* and *Foxp3* genes, respectively. Primer pairs to detect Foxo3 occupancy on the *Foxp3* promoter were as follows (also detailed in Supplementary Table 5):

Conserved non-coding sequence (CNS) 1: For- ACCCTGCAATTATCAGCACAC

Rev- GAAGTGGACTGCTAGAGGGG; CNS3: For- GTTGCCGATGAAGCCCAATG

Rev- TTCCTGCACTGTCTGTTGGG.

Primer pairs to detect Rorγt occupancy on the *Il17a* promoter were as follows: CNS2: For- ATGGGCCTCTCTTTCCACTGATG; Rev-GGAATTTGTGGTGGAAGGGAGTG,

Binding site 2 (BS2): For- GCAAAGCATCTCTGTTCAGCT; Rev- CTTACGTCAAGAGTGGGTTGG; Binding site 3 (BS3): For- GGTACATGTTTGACTGTGCACG; Rev- GTCTTAGACAGCAGTGGTCCT

Threshold cycle (C[T]) values of input DNA were used to calculate percent input of immunoprecipitation, utilizing the following calculation: Percent Input = 2% × 2^(C[T] 2% Input Sample-C[T] IP Sample)^, and the values for the corresponding isotypes were subtracted from the specified antibody used for ChIP. Each reaction was done in triplicate using an Applied Biosystems Step One Plus (ThermoFisher).

### Statistical analysis

All data are representative of at least three independent experiments. Sample sizes were determined using power calculations and are denoted in the figure legends of the respective data. Data is reported as the mean ± standard error of the mean (SEM). Statistical analyzes were performed at a power of 95%, where differences were determined significant when *p* < 0.05. A two-tailed Student’s *t* tests, one-way ANOVA, or two-way ANOVA was performed using the GraphPad Prism Software to determine statistical significance. All ANOVA tests were followed up with a Tukey’s test was used for multiple comparisons assessment with no adjustment. The numerical *p*-value is denoted on graphs. On plots where *p*-value is denoted as asterisks, statistical significance is indicated by *p < 0.05, **p < 0.01, ***p < 0.005, ****p < 0.001.

## Results

### *Gli2*^−/−^ Tregs fail to maintain expression of classical immunosuppressive markers

**Fig. 1:**
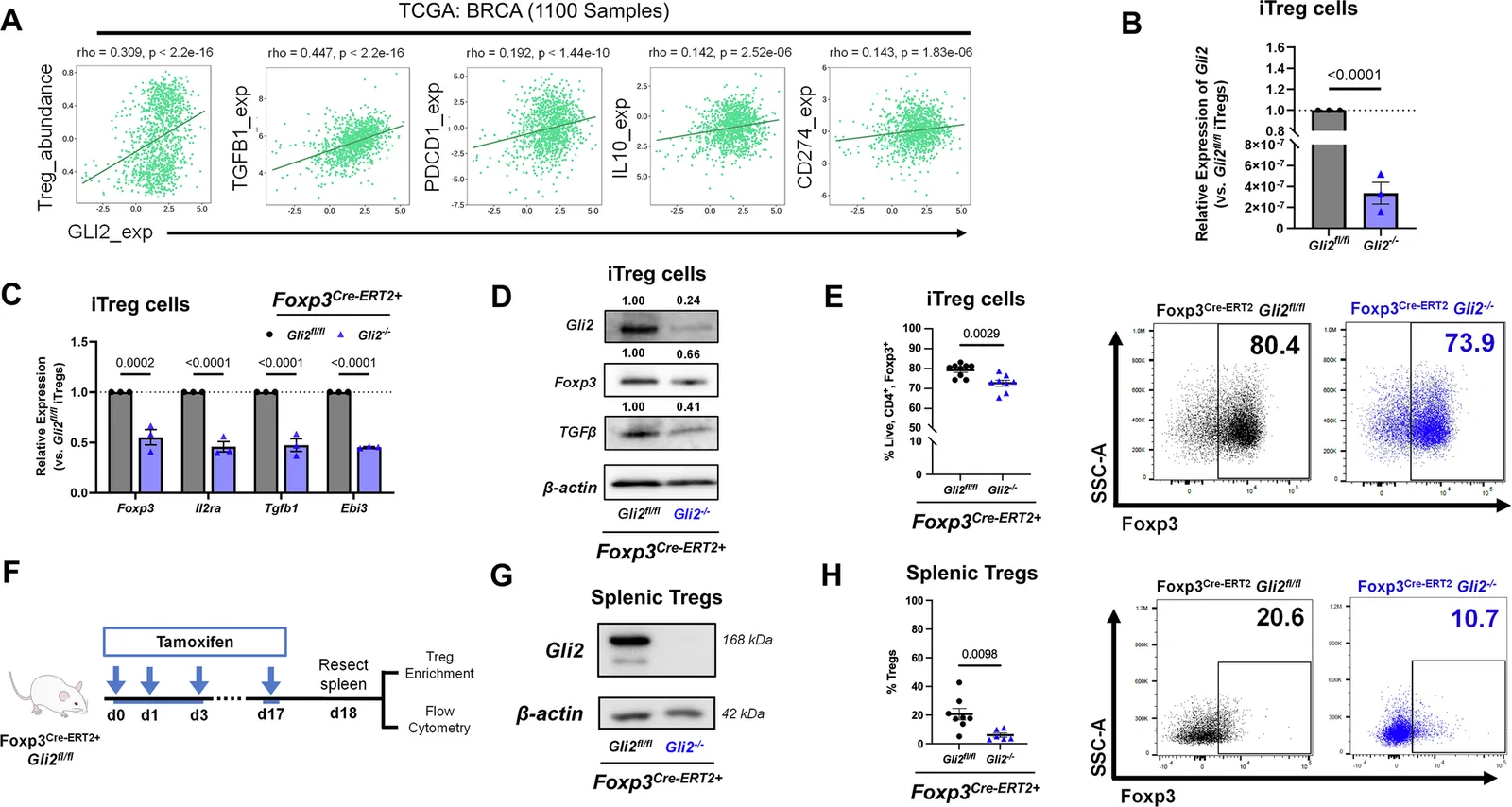
Hh signaling correlates with both immunosuppressive and inflammatory transcriptomes in cancer. **A** The Tumor-immune system interactions database (TISIDB) was utilized to perform analyses to correlate relative *GLI2* expression in human breast tumors with Treg abundance, *TGFB1* (TGF-β), *PDCD1* (PD-1), *IL10*, and *CD274* (PD-L1; left to right). **B** RT-qPCR of relative expression (expressed as 2^^-ΔΔCt^) of *Gli2* in vehicle (*Gli2*^*fl/fl*^) and 4-OHT-treated (*Gli2*^*−/−*^) ex vivo differentiated induced Tregs (iTregs). β-actin was used as the endogenous control. **C** RT-qPCR of relative expression of *Foxp3*, *Il2ra*, *Tgfb1*, and *Ebi3* in *Gli2*^*−/−*^ iTregs versus *Gli2*^*fl/fl*^. **D** Immunoblot of protein expression of Gli2, Foxp3, and TGF-β in *Gli2*^*−/−*^ iTregs. Densitometry was calculated by comparing *Gli2*^*−/−*^ with *Gli2*^*fl/fl*^ iTregs. β-actin was used as loading control. **E** Flow cytometric assessment of Foxp3 expression in *Gli2*^*−/−*^ iTregs with representative dot plots of a control and *Gli2*^*−/−*^ iTreg sample. **F** Experimental design and TAM treatment regimen for characterizing the splenic T cell compartment in Foxp3-*Gli2*^*−/−*^ mice in vivo by western blot, RT-qPCR, and flow cytometry. **G** Immunoblot of Gli2 expression in splenic Tregs enriched from non-tumor-bearing Foxp3-*Gli2*^*−/−*^ (*Gli2*^*−/−*^) and Foxp3-*Gli2*^*fl/fl*^ (*Gli2*^*fl/fl*^) control mice. **H** Flow cytometric assessment of the frequency of splenic Tregs (CD4^+^, Foxp3^+^) with representative dot plots from a control and Foxp3-*Gli2*^*−/−*^ iTreg sample. TISIDB data in correlation plots are representative of 1100 samples from breast cancer patients stored in The Cancer Genome Atlas (TCGA). For flow cytometry, *n* = 9 *Gli2*^*fl/fl*^, 8 *Gli2*^*−/−*^ for ex vivo iTreg analyses and *n* = 8 Foxp3- *Gli2*^*fl/fl*^, 6 Foxp3-*Gli2*^*−/−*^ for in vivo splenic T cell studies.

Hh signaling has been implicated to have a strong influence on tumor immunity, particularly in dampening immunoreactivity in wound healing, atopic dermatitis, pulmonary fibrosis, and other conditions [22, 23, 25–27, 32]. We sought to determine whether Hh activity correlates with Tregs in the TME. We first assessed TISIDB data to evaluate the association between *GLI2* gene expression with Treg abundance and gene expression of key immunosuppressive modulators (*TGFB1, PCDCD1, IL10*, and *CD274*) of the tumor-immune microenvironment (TIME) in breast cancer samples within The Cancer Genome Atlas (TCGA) database [33]. There was a moderately positive correlation of *GLI2* expression with Treg abundance, as well as the expression of the suppressive immunomodulator, *TGFB1*. Additionally, there was a weakly positive correlation between *GLI2* expression and *PDCD1* (PD-1), *IL10*, and *CD274* (PD-L1) expression in the breast tumor TCGA dataset (Fig. 1A). As such, clinical transcriptomic data reveal an intriguing correlation between Gli2 signaling and Tregs in breast cancer.

To uncover the role of Gli2 in Treg biology and tumor immunity, we generated the Foxp3 [CreERT2] *Gli2*^*fl/fl*^ mouse model (Foxp3-*Gli2*^*fl/fl*^), a tamoxifen (TAM)-inducible knockout model that allows for specific ablation of *Gli2* in Tregs (Supplementary Fig. 1A). To validate the successful conditional ablation of *Gli2* in Tregs, naïve splenic CD4^+^ T cells from non-tumor-bearing Foxp3-*Gli2*^*fl/fl*^ mice were induced for differentiation into Tregs (iTregs) and Gli2 ablation was induced with 4-hydroxytamoxifen (4-OHT) treatment for 24 h. A reduction in steady-state transcript levels of *Gli2* RNA was registered in Foxp3-*Gli2*^*−/−*^ iTregs (Fig. 1B). To determine the impact of Gli2 ablation on Tregs, we assessed *Foxp3*, *Il2ra*, *Ebi3*, and *Tgfb1* – all of which are abundantly expressed in Tregs – and registered a reduction in their transcript levels (Fig. 1C). This was solidified by decreased protein levels of Gli2, Foxp3, and TGFβ in Foxp3-*Gli2*^*−/−*^ iTregs (Fig. 1D) and decreased frequency and absolute count of Foxp3^+^ iTregs by flow cytometry (Fig. 1E, and Supplementary Fig. 1B).

To characterize the impact of ablating Gli2 in Tregs in vivo, we treated non-tumor-bearing Foxp3-*Gli2*^*fl/fl*^ mice with TAM (Fig. 1F). Tregs were enriched from the spleens of these non-tumor-bearing Foxp3-*Gli2*^*−/−*^ and control Foxp3-*Gli2*^*fl/fl*^ mice and immunoblotting revealed the successful ablation of the Gli2 protein (Fig. 1G). In parallel, genomic DNA from these splenic Tregs was validated for successful recombination of the *Gli2*^*flox*^ allele (Supplementary Fig. 1C). While the splenic mass from Foxp3-*Gli2*^*−/−*^ mice was more significant as compared Foxp3-*Gli2*^*fl/fl*^ mice (Supplementary Fig. 1D), there was no change in the overall frequency of CD3^+^ T cells and CD4^+^ T cells (Supplementary Fig. 1E, F). However, there was a significant reduction in splenic Tregs in Foxp3-*Gli2*^*−/−*^ mice (Fig. 1H). Thus, in the context of a tumor-naïve mouse, Gli2 signaling in Tregs is essential for maintaining their abundance in the spleen.

### Loss of Gli2 perturbs the stability of Tregs

**Fig. 2:**
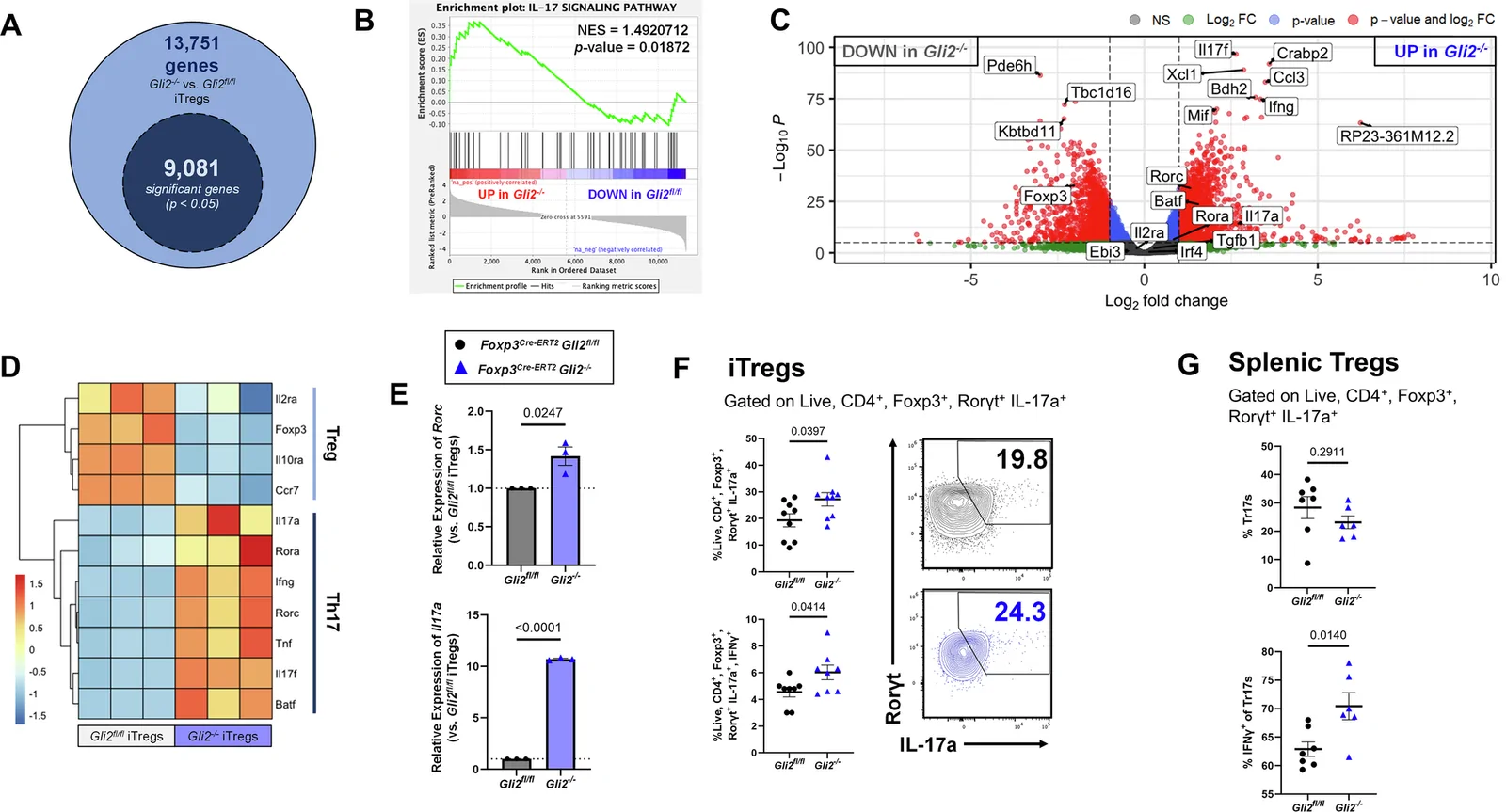
Gli2 ablation in Tregs induces a Tr17 transcriptome and phenotype. Bulk RNA-Seq analysis of *Gli2*^*fl/fl*^ and *Gli2*^*−/−*^ iTregs was performed. **A** The number of DEGs and the number of significant DEGs in the DESeq2 analysis. **B** KEGG pathway analysis results for ‘IL-17 Signaling Pathway’ with the normalized enrichment score and *p*-value. **C** Volcano plot of DEGs up- or downregulated in *Gli2*^*−/−*^ iTregs. **D** Heatmap of a focused Treg-Th17 signature with *Gli2*^*−/−*^ and *Gli2*^*fl/fl*^ iTregs. **E** RT-qPCR of relative expression of Th17-specific markers *Rorc* (left) and *Il17a* (right) in *Gli2*^*fl/fl*^ and *Gli2*^*−/−*^ iTregs. **F** Flow cytometric assessment of double-positive Rorγt^+^ IL-17a^+^ expressing iTregs with representative flow plots, as well as IFNγ^+^ expression in this population when Gli2 is ablated after differentiation. **G** Flow cytometric analysis of overall Tr17 (CD4^+^, Foxp3^+^, Rorγt^+^ IL-17a^+^) and IFNγ^+^ Tr17s (CD4^+^, Foxp3^+^, Rorγt^+^ IL-17a^+^, IFNγ^+^) in spleens of non-tumor-bearing control and Foxp3-*Gli2*^*−/−*^ mice. For flow cytometry, *n* = 9 *Gli2*^*fl/fl*^, 8 *Gli2*^*−/−*^ for ex vivo iTreg analyses and *n* = 7 Foxp3- *Gli2*^*fl/fl*^, 6 Foxp3-*Gli2*^*−/−*^ for in vivo splenic T cell studies. For bulk RNA-Seq, 3 *Gli2*^*fl/fl*^ and 3 *Gli2*^*−/−*^ iTreg biological replicates were sequenced and analyzed.

The deviation in conventional Treg-related molecular read-outs suggested that Gli2 ablation may induce global transcriptomic alterations. Before subjecting to sequencing, the Foxp3-*Gli2*^*fl/fl*^ and Foxp3-*Gli2*^*−/−*^ iTregs were first validated for successful differentiation by assessing Foxp3 expression by RNA levels versus a Th0 control using RT-qPCR (Supplementary Fig. 2A). To identify these changes in the iTreg transcriptome, we performed bulk RNA-Sequencing (RNA-Seq) on Foxp3-*Gli2*^*fl/fl*^ and Foxp3-*Gli2*^*−/−*^ iTregs. Differential gene expression analyzes using *DESeq2* identified 13,751 differentially expressed genes (DEGs) between Foxp3-*Gli2*^*fl/fl*^ and Foxp3-*Gli2*^*−/−*^ iTregs with 9,081 significant DEGs, revealing that Gli2 ablation induces a divergent transcriptome in iTregs (Fig. 2A). Principle component of analysis (PCA) of the three Foxp3-*Gli2*^*fl/fl*^ and three Foxp3-*Gli2*^*−/−*^ iTreg samples illustrates the transcriptomic alterations (Supplementary Fig. 2B, C). Pathway analysis using the Kyoto Encyclopedia of Genes and Genomes (KEGG) reference database was performed using the significant DEGs found between the Foxp3-*Gli2*^*fl/fl*^ and Foxp3-*Gli2*^*−/−*^ iTreg samples. In this unbiased pathway analysis, the IL-17 signaling pathway emerged as one of the top enriched immune-relevant pathways upregulated in Gli2-ablated iTregs (Fig. 2B, and Supplementary Fig. 2D). Therefore, a more in-depth analysis of Th17 and IL-17-associated genes was conducted. Indeed, the top upregulated DEGs in Foxp3-*Gli2*^*−/−*^ iTregs versus control include Th17-relevant genes (*Il17a, Il17f, Rorc, Rora, Batf, Irf4*, *Ifng*), while genes characteristic of Tregs, such as *Foxp3* and *Il2ra*, were downregulated in Foxp3-*Gli2*^*−/−*^ iTregs (Fig. 2C, and Supplementary Table 2). In fact, a focused assessment of conventional Treg (*Foxp3, Il2ra, Il10ra, Ccr7*) and inflammatory Th17 signatures (*Rorc, Rora, Il17a, Il17f, Ifng, Tnf, Batf*) revealed that Foxp3-*Gli2*^*−/−*^ iTregs align more with the Th17 signature than the Treg signature, cumulatively revealing a composite transcriptome that holds properties of a Tr17 profile (Fig. 2D). In iTregs, this profile was substantiated by an increase in the steady-state transcript levels of Th17-specific markers, *Rorc* and *Il17a* as assessed by RT-qPCR (Fig. 2E). We next assessed Tr17 and Th17 cell populations utilizing the gating strategy detailed in Supplementary Fig. 2E, F. Flow cytometric analysis of Foxp3-*Gli2*^*−/−*^ iTregs revealed a significant increase in Foxp3^+^ cells co-expressing Rorγt and IL-17a (Tr17 cells) and in IFNγ-expressing Tr17 cells (inflammatory Tr17 cells) as compared to control, suggesting that Gli2 ablation spurs the transition of Tregs to inflammatory Tr17 cells (Fig. 2F, and Supplementary Fig. 2G). While the frequency of splenic Tr17 and Th17 cells remained comparable in unchallenged Foxp3-*Gli2*^*−/−*^ mice, there was an increase in IFNγ-expressing Tr17 and Th17 cells (Fig. 2G, and Supplementary Fig. 2H), illustrating the acquisition of inflammatory attributes by Tregs.

### Hh inhibition programs Tregs to assume attributes of composite Tr17 cells

**Fig. 3:**
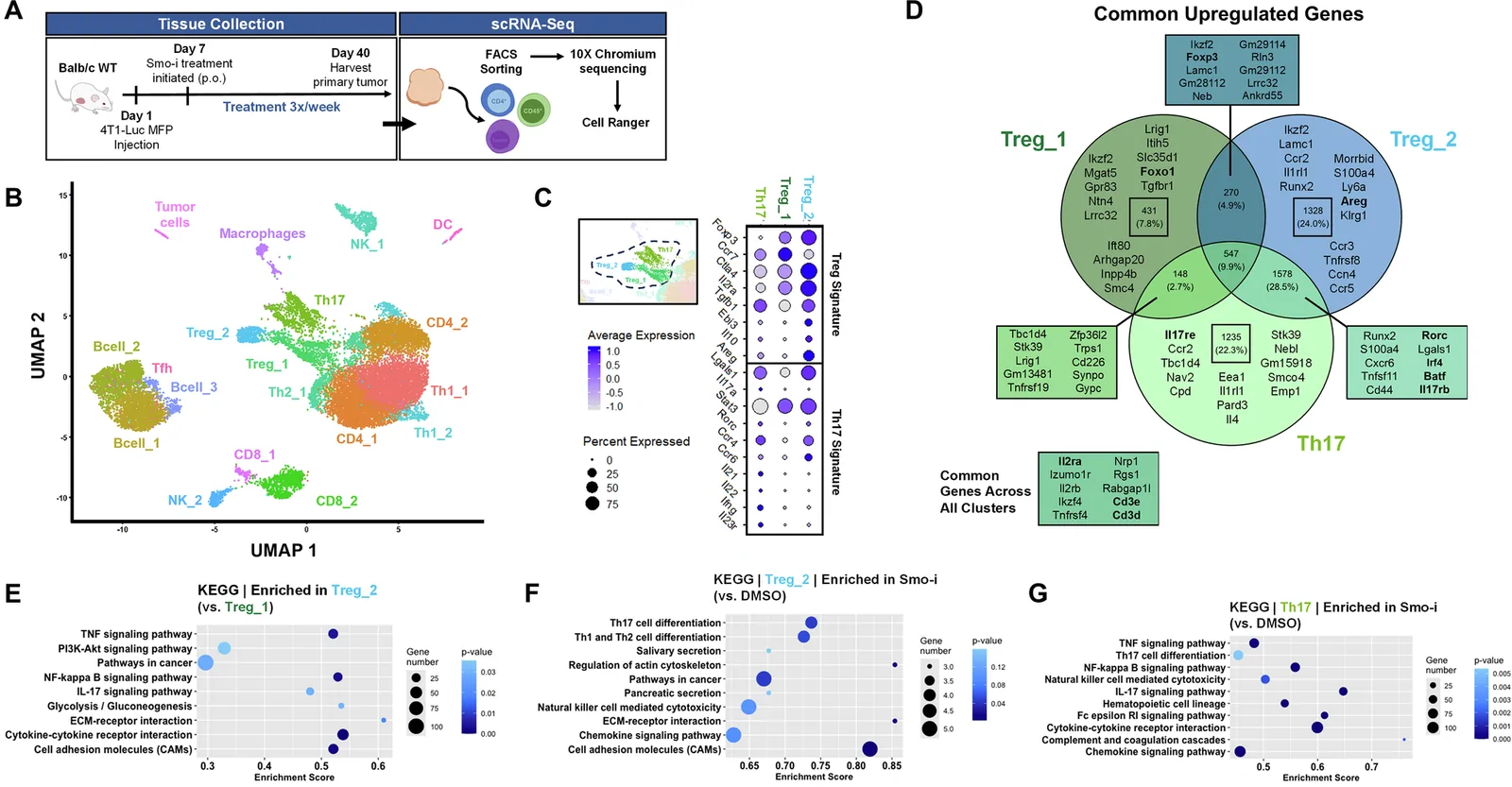
There are distinct Treg transcriptomes in the 4T1 murine mouse model. Primary 4T1 mammary tumors harvested from wildtype Balb/c mice treated with Smo-i or vehicle (DMSO) were subjected to scRNA-Seq using the 10X Chromium platform. **A** A schematic of the experimental design for the 4T1 mouse model and pharmacologic Smo-i treatment regimen. **B** Representative UMAP of clusters present in the scRNA-Seq dataset. **C** Representative UMAP and dot plot of Treg- and Th17-relevant genes across the Treg and Th17 clusters in the dataset. **D** Venn diagram illustrating the top common upregulated DEGs between the Treg_1, Treg_2, and Th17 clusters. **E** Top enriched KEGG pathways in Treg_2 vs. Treg_1 clusters. **F** Top enriched KEGG pathways in Smo-i Treg_2 versus DMSO Treg_2 clusters. **G** Top enriched pathways in Smo-i Th17s versus DMSO Th17s. For scRNA-Seq analysis, *n* = 2 tumor per condition used for scRNA-Seq differential gene expression analysis.

In the context of a mammary tumor, systemic Hh inhibition resulted in a paradigm shift involving the transition of Tregs to the Tr17 phenotype, contributing to an inflammatory TME [22]. This evidence warranted an assessment of major alterations in the whole tumor transcriptome when Hh signaling is systemically inhibited. We utilized the single-cell RNA Sequencing (scRNA-Seq) dataset generated from the 4T1 syngeneic (Balb/c) tumors that were treated with Smo-i (or vehicle control; Fig. 3A). Feature-barcode matrices were generated using the Cell Ranger *count* pipeline and scRNA-Seq analysis was conducted using Seurat v5. In this analysis, the resolution was identified by assessing the most optimal principal components that significantly capture the complexity of the Treg and Th17 clusters within the dataset, which identified two divergent Treg clusters. Top DEGs of each cluster were used to annotate and assign the cell type identities for the T cells, tumor cells, and other immune cell populations present in the dataset (Fig. 3B, and Supplementary Table 2). Common Treg and Th17 markers (*Il2ra*, *Foxp3*, *Rorc*, and *Il17a*) were used to identify the Treg and Th17 clusters (Supplementary Fig. 3A). Following Seurat’s graph-based clustering, two Treg clusters were identified (labeled as “Treg_1” and “Treg_2”). Comparative analyzes were performed to contrast the Treg clusters to the Th17 cluster present in the dataset to determine if any of the Treg clusters express a Tr17/Th17 transcriptome. It was intriguing to note that despite sharing a prominent Treg gene signature with the Treg_1 cluster, the Treg_2 cluster had an overlapping Th17 gene signature with the Th17 cluster (Fig. 3C). When comparing the two Treg clusters alone, the top DEGs upregulated in Treg_2 as compared to Treg_1 were genes associated with inflammatory activity, such as *Icos*, *Itgae*, and *Itgav*; concordantly, Treg-relevant genes such as *Bach2*, *Ccr7*, and *Foxp1* were downregulated (Supplementary Fig. 3B). Next, all shared and unique up- and downregulated genes were compared across the Treg_1, Treg_2, and Th17 clusters. This analysis revealed 547 upregulated genes that were shared across all three clusters, including *Cd3e*, *Cd3d*, and *Il2ra*. When comparing upregulated genes shared between Treg_2 and Th17 clusters but not in the Treg_1 cluster, genes involved in the Th17 phenotype, such as *Rora*, *Batf*, and *Irf4* were shared between both clusters. Despite these common upregulated Th17-relevant genes, the Treg_2 cluster also shared the prominent common Treg-relevant *Foxp3* gene with Treg_1 (Fig. 3D). Notably, commonly downregulated genes between the Th17 and Treg_2 clusters included Treg-promoting genes, such as *Foxp1* and *Ccr7* (Supplementary Fig. 3C) [39]. KEGG pathway analysis of the two Treg clusters also revealed an enrichment of positive regulators of the IL-17 signaling pathway in the Treg_2 cluster (Fig. 3E, and Supplementary Fig. 3D). In addition to IL-17 signaling, key genes associated with other inflammatory pathways, including NF-κB and TNFα signaling, were also enriched in the Treg_2 and Th17 clusters (Supplementary Fig. 3E, F) [17, 18]. Given the attributes of a prominent Treg and Th17 signature, the Treg_2 cluster was identified as a Tr17 population.

To assess the influence of Hh inhibition on the transcriptomic profile of this distinctive Tr17 (Treg_2) cluster, KEGG pathway analysis was performed using DEGs between the Treg_2 cluster in Smo-i- and DMSO-treated tumors. The ‘Th17 cell differentiation’ as well as other inflammatory-related processes were amongst the top significantly enriched pathways in the Smo-i Treg_2 cluster compared to control (Fig. 3F). This data provided transcriptomic evidence that Hh inhibition can spur Treg-Tr17 transition. Furthermore, subjecting the Th17 cluster to KEGG analysis identified enrichment of “IL-17 signaling”, “TNF signaling”, and “Th17 cell differentiation” pathways in the Smo-i-treated Th17 cluster (Fig. 3G). As such, we identified the Treg cluster that assumed attributes of composite inflammatory Tr17 cells likely spurring an enrichment of Th17 cells upon systemic Hh inhibition.

### Gli2 critically regulates immunosuppressive activity of Tregs

**Fig. 4:**
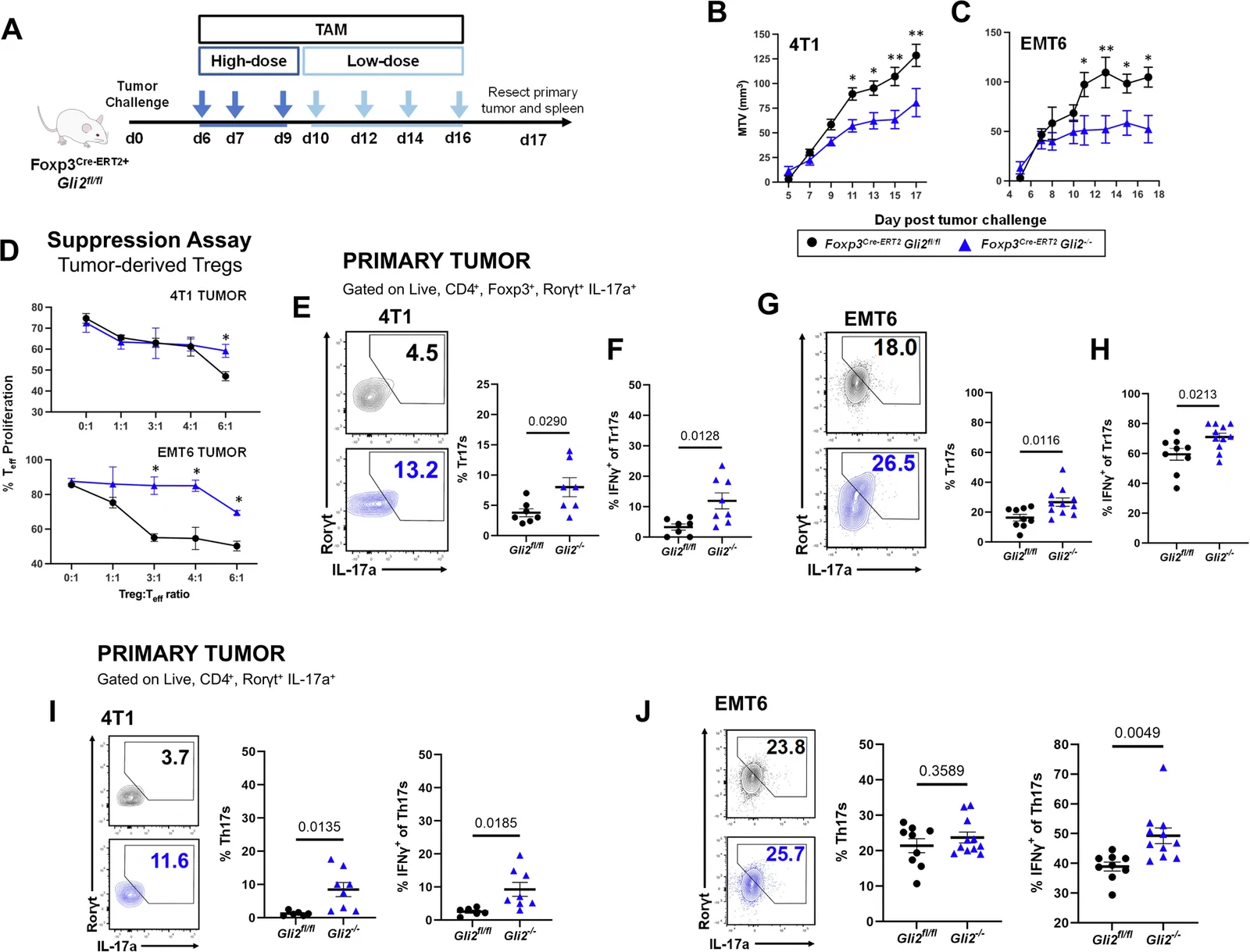
Loss of Gli2 impairs the immunosuppression of tumoral Tregs and induces their transition to inflammatory Tr17s. **A** Schematic of the experimental design for assessing Treg activity by inducing Gli2 ablation in vivo in 4T1 Foxp3-*Gli2*^*fl/fl*^ mice after tumor establishment. Average tumor growth of **B** 4T1 or **C** EMT6 Foxp3-*Gli2*^*fl/fl*^ and Foxp3-*Gli2*^*−/−*^ mice throughout the duration of the study. Tumor growth is expressed as mean tumor volume (MTV). **D** Treg suppression assay data expressed as percent proliferation of the CD4^+^ T_eff_ cell populations as assessed by CFSE staining acquired by flow cytometry. For suppression assay, Tregs were derived from the tumors of 4T1 Foxp3-*Gli2*^*−/−*^ (top), EMT6 Foxp3-*Gli2*^*−/−*^ (bottom), and the appropriate control mice after the Treg activity TAM treatment regimen. **E** Flow cytometry analysis of overall Tr17s (CD4^+^, Foxp3^+^, Rorγt^+^ IL-17a^+^) in the primary tumor of 4T1 Foxp3-*Gli2*^*fl/fl*^ and Foxp3-*Gli2*^*−/−*^ mice with representative contour plots. **F** Flow cytometry analysis of IFNγ^+^ Tr17 cells and IFNγ^+^ Th17 cells in the primary tumors of 4T1 Foxp3-*Gli2*^*fl/fl*^ and Foxp3-*Gli2*^*−/−*^ mice. **G** Flow cytometry analysis of overall Tr17s (CD4^+^, Foxp3^+^, Rorγt^+^ IL-17a^+^) in the primary tumor of EMT6 Foxp3-*Gli2*^*fl/fl*^ and Foxp3-*Gli2*^*−/−*^ mice with representative contour plots. **H** Flow cytometry analysis of IFNγ^+^ Tr17 cells and IFNγ^+^ Th17 cells in the primary tumors of EMT6 Foxp3-*Gli2*^*fl/fl*^ and Foxp3-*Gli2*^*−/−*^ mice. **I** Flow cytometry analysis of overall and IFNγ^+^ Th17 cells in the primary tumors of 4T1 Foxp3-*Gli2*^*fl/fl*^ and Foxp3-*Gli2*^*−/−*^ mice. **J** Flow cytometry analysis of overall and IFNγ^+^ Th17 cells in the primary tumors of EMT6 Foxp3-*Gli2*^*fl/fl*^ and Foxp3-*Gli2*^*−/−*^ mice. For flow cytometry studies, *n* = 7–9 Foxp3-*Gli2*^*fl/fl*^, 6-10 Foxp3-*Gli2*^*−/−*^. Data representative of two repeats. Cytokine array data is representative of *n* = 3 Foxp3-*Gli2*^*fl/fl*^, 3 Foxp3-*Gli2*^*−/−*^ 4T1 tumor lysates. For tumor growth and Treg suppression plots, *p < 0.05, **p < 0.01.

Informed by these analyzes, we next assessed the impact of ablating Gli2 in Tregs on their activity. To accomplish this, Foxp3-*Gli2*^*fl/fl*^ mice were tumor challenged with 4T1 or EMT6 triple-negative mammary carcinoma cells and treated with TAM upon tumor establishment as depicted in Fig. 4A. Gli2 ablation resulted in a significant reduction in tumor growth in both the 4T1 and EMT6 models (Fig. 4B, C). There was a trending decrease in splenic and tumoral mass in Foxp3-*Gli2*^*−/−*^ mice in both models at termination (Supplementary Fig. 4A–D). In the 4T1 model, there was no change in tumoral CD4^+^ T cells with increased splenic CD4^+^ T cells. In the EMT6 model, there was a decrease in tumoral CD4^+^ T cells from Foxp3-*Gli2*^*−/−*^ mice, while the splenic CD4^+^ T cells remained unaltered (Supplementary Fig. 4E, F). Interestingly, there was a significant decrease in tumoral and splenic Tregs in Foxp3-*Gli2*^*−/−*^ mice in the 4T1 model, with no change in the EMT6 model (Supplementary Fig. 4G–J). We next assessed whether Gli2 ablation in Tregs impacts their immunosuppressive activity and the proliferation of target effector T cells (T_eff_). A Treg suppression assay was performed using tumor- or spleen-derived Tregs from tumor-bearing Foxp3-*Gli2*^*−/−*^ or Foxp3-*Gli2*^*fl/f*^ mice. Tregs were co-cultured with CFSE-labeled naïve T_eff_ CD4^+^ T cells at differing ratios. Proliferation of T_eff_ cells was used as a readout of Treg suppression. In both the 4T1 and EMT6 tumor model systems, tumor-derived Tregs from Foxp3-*Gli2*^*−/−*^ mice exhibited decreased immunosuppression, as denoted by the higher proliferation of T_eff_ cells (Fig. 4D). This data suggests that Gli2 ablation invoked a prominent impact on tumoral Tregs, highlighting an essential role of Gli2 in modulating Treg function in the TME.

Variations between the 4T1 and EMT6 models are likely driven by the differential characteristics between the two cell lines. Compared to 4T1 cells, EMT6 tumor cells secrete higher levels of TGFβ, a signaling superfamily that holds diverse, context-dependent functions in Treg and Th17 biology [40, 41]. As such, this may influence the distinct effects of Gli2 ablation on Tregs between these model systems. The differences in the immunogenicity between the 4T1 and EMT6 TNBC models may also contribute to the unique findings noted between both model systems [42].

We next sought to characterize changes in the TME in the context of Treg-specific Gli2 ablation. Cytokine profiling of 4T1 tumors revealed a prominent decrease in several Treg-relevant cytokines and chemokines in tumors from Foxp3-*Gli2*^*−/−*^ mice. There was a decrease in IL-2 and IL-10, interleukins highly involved and essential for Treg function during tumor response (Supplementary Fig. 4K) and a significant reduction in Ccl11, Ccl22, Cxcl13, and Ccl17, all of which are involved in Treg retention within the TME in breast and other cancer types (Supplementary Fig. 4L) [43–46]. To assign clinical relevance to these findings, we queried the correlative expression of these cytokines and *GLI2* in breast cancer samples from TISIDB and determined that all these cytokines are positively correlated with *GLI2* expression (Supplementary Fig. 4M), further suggesting that ablation of Gli2 in Tregs perturbs immune-suppressive cytokines in the tumor.

Informed by our bulk RNA-seq analysis (Fig. 2) and scRNA-Seq data (Fig. 3), we assessed the impact of Gli2 ablation on Tr17 and Th17 cells. Compared to tumors from Foxp3-*Gli2*^*fl/fl*^ mice, the 4T1 and EMT6 mammary tumors from Foxp3-*Gli*^*−/−*^ mice registered a significant increase in the abundance of Tr17 cells and inflammatory IFNγ^+^ Tr17 and IFNγ^+^ Th17 cells (Fig. 4E–H). There was an increase in overall Th17 cells in 4T1 mammary tumors from Foxp3-*Gli2*^*−/−*^ mice, but there was no change in the EMT6 mammary tumors. However, there was an increased frequency of tumoral IFNγ^+^ Th17 cells in both models (Fig. 4I, J). We also registered an increase in both Tr17 and Th17 populations in the spleens of Foxp3-*Gli2*^*−/−*^ mice in both models. In the 4T1 model, there was an increase in IFNγ^+^ Tr17 and IFNγ^+^ Th17 populations, while there is no change in IFNγ^+^ expression in these populations in the EMT6 model (Supplementary Fig. 4N, O). Collectively, these data demonstrate that Gli2 critically regulates immunosuppressive activity and provokes an inflammatory profile in Tregs in vivo.

### Gli2 loss disrupts Foxo3 and promotes Rorγt transcriptional activity in Tregs

Thus far, the data show that Gli2 ablation impairs the immunosuppressive activity of Tregs and elicits acquisition of features of inflammatory Th17 cells. Next, through in-depth molecular studies, we sought to investigate if Gli2 influences the transcription factors Foxp3 and/or Rorγt. Forkhead box O (Foxo) genes are important in Treg differentiation by virtue of their role in regulating TGF-β-induced Foxp3 expression. Specifically, Foxo1 and Foxo3 control Foxp3 promoter activity by binding the Foxp3 locus at sites in the conserved noncoding sequences (CNS) 1 and 3 [47]. Publicly available ChIP-Sequencing data from human acute monocytic leukemic THP-1 cells (GSM2212253) suggest that GLI2 binds to the regulatory regions of Foxo1 (Supplementary Fig. 5A, B). Therefore, we sought to determine if Gli2 ablation disrupts Foxo1 and Foxo3 activity, and consequently, alters the regulatory role of these proteins on Foxp3.

**Fig. 5:**
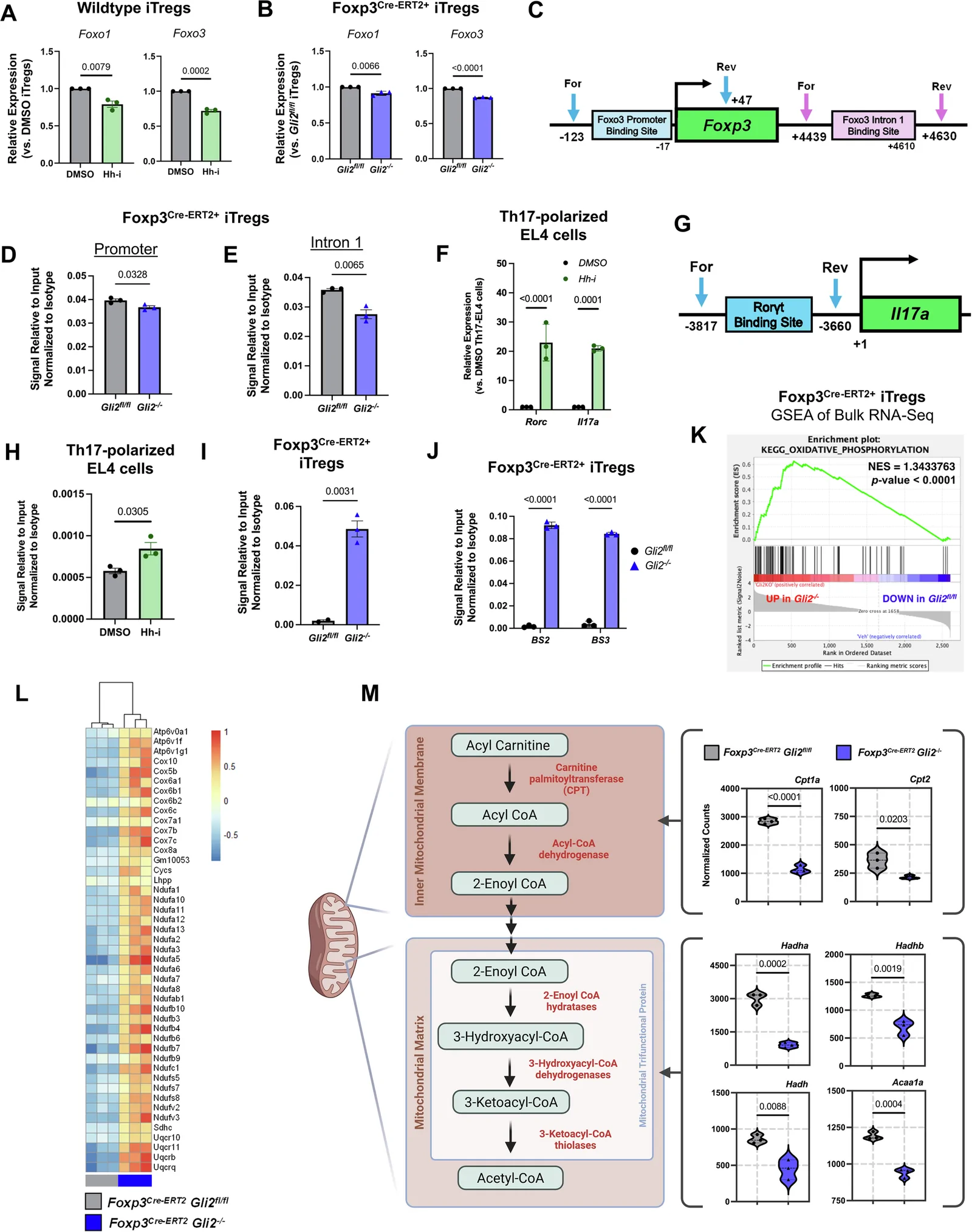
Gli2 deletion disrupts Foxo3 activity and promotes Rorγt binding to *Il17a.* **A** RT-qPCR of *Foxo1* and *Foxo3* using iTregs treated with GANT61 (Hh-i) or vehicle control. **B** RT-qPCR of *Foxo1* and *Foxo3* in *Gli2*^*fl/fl*^ and *Gli2*^*−/−*^ iTregs. **C** Primer design for assessing Foxo3 binding capacity to *Foxp3* via ChIP. Arrows indicate the location of the forward (For) and reverse (Rev) primers used for ChIP-qPCR. ChIP qPCR of Foxo3 (**D**) promoter and **E** intron 1 binding sites in the *Foxp3* gene in *Gli2*^*fl/fl*^ and *Gli2*^*−/−*^ iTregs. Each reaction was done in triplicate. **F** RT-qPCR of *Rorc* and *Il17a* using Th17-polarized EL4s (Th17-EL4s) treated with Hh-i or vehicle control. **G** Primer design and schematic representation of Rorγt binding to the *Il17a* gene in the *CNS2* region. Rorγt binds to a consensus binding sequence ~3800 bp upstream of the Il17a promoter. Arrows indicate the location of the forward (For) and reverse (Rev) primers used for ChIP-qPCR. **H** ChIP-qPCR of Rorγt-binding to the *Il17a* promoter region in Th17-EL4s treated with or without Hh-i. **I** ChIP-qPCR of Rorγt binding at the CNS2 regulatory sites in the *Il17a* promoter in *Gli2*^*fl/fl*^ and *Gli2*^*−/−*^ iTregs. **J** ChIP qPCR of Rorγt binding site 2 (BS2) regulatory site and binding site 3 (BS3) regulatory site in the *Il17a* promoter in *Gli2*^*fl/fl*^ and *Gli2*^*−/−*^ iTregs. **K** KEGG pathway enrichment analysis for ‘Oxidative Phosphorylation’ with the normalized enrichment score and *p*-value. **L** Heatmap of genes involved in oxidation phosphorylation that are significantly altered in *Gli2*^*−/−*^ iTregs versus control in the bulk RNA-Seq dataset. **M** Schematic and normalized counts of FAO-relevant genes (*Cpt1a, Cpt2, Hadh, Hadha, Hadhb*, and *Acaa1a*) in the *Gli2*^*fl/fl*^ and *Gli2*^*−/−*^ iTregs bulk RNA-Seq dataset. All ChIP-qPCR data is represented as percent of input relative to IgG control. For bulk RNA-Seq, 3 *Gli2*^*fl/fl*^ and 3 *Gli2*^*−/−*^ iTreg biological replicates were sequenced and analyzed.

We first assessed *Foxo1* and *Foxo3* transcript levels in iTregs inhibited for Hh activity with Smo-i or GANT61 (Hh-i). GANT61 is a Gli antagonist that inhibits the ability of Gli proteins to bind DNA [48]. There was a reduction in the steady-state transcript levels of *Foxo1* and *Foxo3* in iTregs treated with either Hh-i or Smo-i as compared to the vehicle (DMSO) control (Fig. 5A, and Supplementary Fig. 5C, D). This was affirmed when we queried the steady-state transcript levels of *Foxo1* and *Foxo3* in *Gli2*^*fl/fl*^ and *Gli2*^*−/−*^ iTregs (Fig. 5B). A decrease in *Foxo3* but not *Foxo1* was noted in the bulk RNA-Seq analysis of Foxp3-*Gli2*^*−/−*^ iTregs compared to Foxp3-*Gli2*^*fl/fl*^ iTregs (Supplementary Fig. 5E).

Informed by this evidence, we assessed the occupancy of Foxo3 at the regulatory sites of *Foxp3*, including its promoter and in intron 1 sites, using chromatin immunoprecipitation (ChIP) followed by RT-qPCR in Foxp3-*Gli2*^*fl/fl*^ and Foxp3-*Gli2*^*−/−*^ iTregs (Fig. 5C). There was a significant reduction in the occupancy of Foxo3 at the promoter and in intron 1 of *Foxp3* in the Foxp3-*Gli2*^*−/−*^ iTregs as compared to Foxp3-*Gli2*^*fl/fl*^ iTregs (Fig. 5D, E), indicating the ability of Foxo3 to transcriptionally regulate *Foxp3* is disrupted by Gli2 ablation, contributing to impaired Foxp3 levels.

As previously highlighted, Rorγt and IL-17 are essential for differentiation of Th17 cells [49]. We next investigated if inhibition of Gli activity in Tregs influences the transcriptional program of Th17 differentiation. For this, we utilized EL4 cells as a model for Th17 differentiation. TCR stimulation by αCD3/CD28 induces the expression of IL-17a and Rorγt, polarizing EL4s to exhibit a Th17 phenotype [50–52]. To assess if Hh inhibition influences *IL17a* and *Rorc* expression, EL4 cells were skewed to Th17 cells, followed by treatment with Hh-i for 24 h. In response to Hh-i, there was an increase in *Rorc* and *Il17a* in Th17-skewed EL4s (Fig. 5F).

To initiate the Th17 differentiation program, Rorγt binds to ROR response elements in the CNS2 locus within the *Il17a* regulatory region [51, 52]. Binding of Rorγt to CNS2 initiates a cascade of events—CNS2 associates with the *IL17a* promoter via a chromatin loop and leads to the activation of *IL17a* transcription following the recruitment of histone-modifying enzymes [53, 54]. To investigate if the binding of Rorγt to CNS2 within the *Il17a* regulatory region is influenced by Hh inhibition, we assessed the occupancy of Rorγt at the CNS2 region of *Il17a* by ChIP-RT-qPCR in Th17-polarized EL4 cells in the context of Hh-i for 24 h (Fig. 5G). There was enhanced binding of Rorγt at CNS2 of the *Il17a* promoter region in Hh-i-treated Th17-EL4 cells (Fig. 5H). To assess if Gli2 ablation in Tregs elicits occupancy of Rorγt at the CNS2 locus of the *Il17a* promoter, we repeated the ChIP-qPCR using *Gli2*^*fl/fl*^ and *Gli2*^*−/−*^ iTregs. Similar to Hh-i-treated Th17-skewed EL4 cells, there was a stark increase in the occupancy of Rorγt at the CNS2 site in the *Il17a* promoter region (Fig. 5I). In addition to the site that we queried, we also affirmed that in Foxp3-*Gli2*^*−/−*^ iTregs, there was increased occupancy of Rorγt at two additional binding sites (BS2 and BS3) in the *Il17a* promoter (Fig. 5J) [54]. Altogether, the data demonstrate that in the absence of Gli2, the ability of Foxo3 to aid in Foxp3 expression and subsequent Treg differentiation is disrupted.

### Gli2 ablation induces a metabolic transition associated with Treg plasticity

Metabolic reprogramming plays an essential role in the transition of cell states, especially in Treg stability and plasticity [54]. Oxidative phosphorylation (OXPHOS) plays a key role in regulating the plasticity of Tregs to transition to Th17 cells [55, 56] while fatty acid oxidation (FAO) is essential for Treg function and immunosuppression [57, 58]. In agreement with this, GSEA of bulk RNA-Seq from *Gli2*^*fl/fl*^ and *Gli2*^*−/−*^ iTregs revealed that OXPHOS was enriched in Foxp3-*Gli2*^*−/−*^ iTregs (Fig. 5K, and Supplementary Fig. 2D). This is further supported by upregulation of genes associated with the subunits of complexes involved in cellular respiration and the mitochondrial electron transport chain components, such as cytochrome c oxidase and ubiquinone (Fig. 5L) [58].

Next, we assessed the relative gene expression of enzymes associated with key steps in mitochondrial FAO. In Foxp3-*Gli2*^*−/−*^ iTregs, there was a significant reduction in *Cpt1a* (carnitine palmitoyltransferase 1A), *Cpt2*, *Hadh* (3-hydroxyacyl-CoA dehydrogenase) and its subunits (*Hadha* and *Hadhb*), and *Acaa1a* (acetyl-CoA Acyltransferase 1) (Fig. 5M). Altogether, Gli2 ablation promoted binding of Rorγt to the CNS2 regulatory region of *IL17a*, potentially orchestrating prominent metabolic changes in metabolism, thereby pivoting the regulatory program in Tregs.

### Spatial transcriptomics reveals that Treg-specific Gli2 ablation promotes an inflammatory tumor niche

To evaluate the effects of Gli2 ablation in Tregs on the transcriptome and spatial biology of the TME, we performed spatial transcriptomics on 4T1 tumors harvested from Foxp3-*Gli2*^*fl/fl*^ and Foxp3-*Gli2*^*−/−*^ mice using the 10X Visium V1 platform (Supplementary Fig. 6A). Foxp3-*Gli2*^*fl/fl*^ and Foxp3-*Gli2*^*−/−*^ tumors were annotated and analyzes of only the identified tumor regions were spatially barcoded and subjected to spatial analyzes (Supplementary Fig. 6B, C). One of the Foxp3-*Gli2*^*fl/fl*^ control tumor tissues unintentionally contained a tumor-draining lymph node and one of the Foxp3-*Gli2*^*−/−*^ tumors had residual muscle tissue—both were excluded from the analysis. A total of nine clusters were identified in the analysis of the heterogeneous tumor landscape utilizing top DEGs of each cluster (Supplementary Table 3) [59]. Given the resolution of the Visium V1 technology, transcriptional signatures were used to annotate each cluster using top DEGs observed, revealing and deconvoluting tumor-, macrophage-, and immune cell-relevant signatures that represented the various TME constituents (Supplementary Fig. 6D). Applying tumor- (*Epcam, Krt7, Krt8, Krt14*), macrophage- (*Adgre1, Mrc1, Cd163, Il4ra, Stat1*), B cell- (*Cd19, Cd20*), and T cell-focused (*Cd3e, Cd4, Cd8a, Foxp3*) signatures allowed the characterization of the dataset and the identification of four zones with unique composition of these prominent cell populations: “Immune-enriched”, “Tumor_Macrophage-enriched”, “Tumor-enriched”, and “Tumor_Immune-enriched” (Supplementary Fig. 6E–H). A representative UMAP of the nine clusters revealed that cluster 0 represents the zone enriched in tumor cell signatures (“Tumor”). Clusters 1, 5, and 6 represented the tumor and macrophage enriched zone (“Tumor_Macrophage”). Clusters 2, 4, and 7 represented the zone enriched in innate and adaptive immune cell signatures (“Immune”). Lastly, clusters 3 and 8 represented the zone enriched in both tumor and immune cell-related signatures. These signatures were present across all four tumors within the dataset and were utilized to annotate the predominant populations present within each cluster (Supplementary Fig. 7A, B). The enrichment of immune, tumor, and macrophage populations was also confirmed by applying spatial dimensional plots for *Ptprc*, *Epcam*, and *Mrc1*, respectively (Supplementary Fig. 7D–F). These zones were identified and uniquely mapped to distinct locations in the TME (Supplementary Fig. 7G). The tumor-macrophage signature-enriched zones lie more central to the bordering immune zone of the tumors. Both zones exhibited an enrichment of macrophages supported by transcriptional signatures, consistent with prior studies showing that macrophages are a highly represented immune population that often borders tumor edges. Macrophages represent around 40% of immune cells in breast tumors, including mouse mammary tumors [60]. The “Tumor” enriched zone dominates the central region of the tumor and neighbors a less abundant “Tumor-immune” enriched zone (Supplementary Fig. 8A, B). The WebGestalt platform was used to further characterize each zone by performing gene ontology (GO) analyzes to identify biological processes [37]. The “Immune” and “Tumor” enriched zones had an enrichment of GO terms specific to immune response-related biological processes, whereas the “Tumor-Macrophage” and “Tumor-Immune” enriched zones had a significant enrichment of GO terms associated with cell cycle, maintenance, and angiogenesis (Supplementary Fig. 8C–F). Altogether, we annotated and identified regions of the heterogeneous TME with distinctive transcriptomes and spatial relationships.

With the heterogeneous TME now annotated based on distinctive transcriptomes and spatial relationships of each zone, we next explored transcriptomic changes in these zones between mammary tumors from Foxp3-*Gli2*^*fl/fl*^ and Foxp3-*Gli2*^*−/−*^ mice. We applied deconvolution and identification of zones to assess transcriptomic and spatial modifications in designated areas of the tumor induced by the modified function of *Gli2*^*−/−*^ Tregs. This allowed us to overcome the limited resolution in capturing Treg populations due to technological limitations (Supplementary Fig. 9A). Although the spatial mapping of the identified clusters and zones was similar between tumors from Foxp3-*Gli2*^*fl/fl*^ and Foxp3-*Gli2*^*−/−*^ mice (Fig. 8A-B 6F), there were differences in the proportion of clusters and zones in the tumors from Foxp3-*Gli2*^*−/−*^ mice. Cluster 3, a part of the “Tumor_Immune” enriched zone, was not abundantly present in the Foxp3-*Gli2*^*−/−*^-derived tumors (20.8% vs. 0.1%); however, only one of the Foxp3-*Gli2*^*fl/fl*^-derived tumors contributed to the abundance of cluster 3 (Supplementary Fig. 9B, C). Specifically with regards to the “Tumor” enriched zone (cluster 0), Foxp3-*Gli2*^*−/−*^-derived tumors held a larger fraction of this zone (13.5% vs. 28.4%). The “Tumor_Macrophage” enriched zone was also more prominent in tumors from Foxp3-*Gli2*^*−/−*^ tumors as compared to tumors from Foxp3-*Gli2*^*fl/fl*^ mice (29.5% vs. 41.4%). Both control and Foxp3-*Gli2*^*−/−*^ tumors had a similar portion of the “Immune-enriched” zone (34.4% vs. 28.2%). Due to the lack of spots in cluster 3, the “Tumor-Immune” enriched zone only represents 1.9% out of all spots in Foxp3-*Gli2*^*fl/fl*^ tumors (Supplementary Fig. 9D–F). As shown previously, the “Tumor” enriched zone also had a significant enrichment of GO biological processes involved in cancer cell apoptosis and the activation of immune response, including interferon-alpha and interferon-beta response (Supplementary Fig. 8E 9J).

**Fig. 6:**
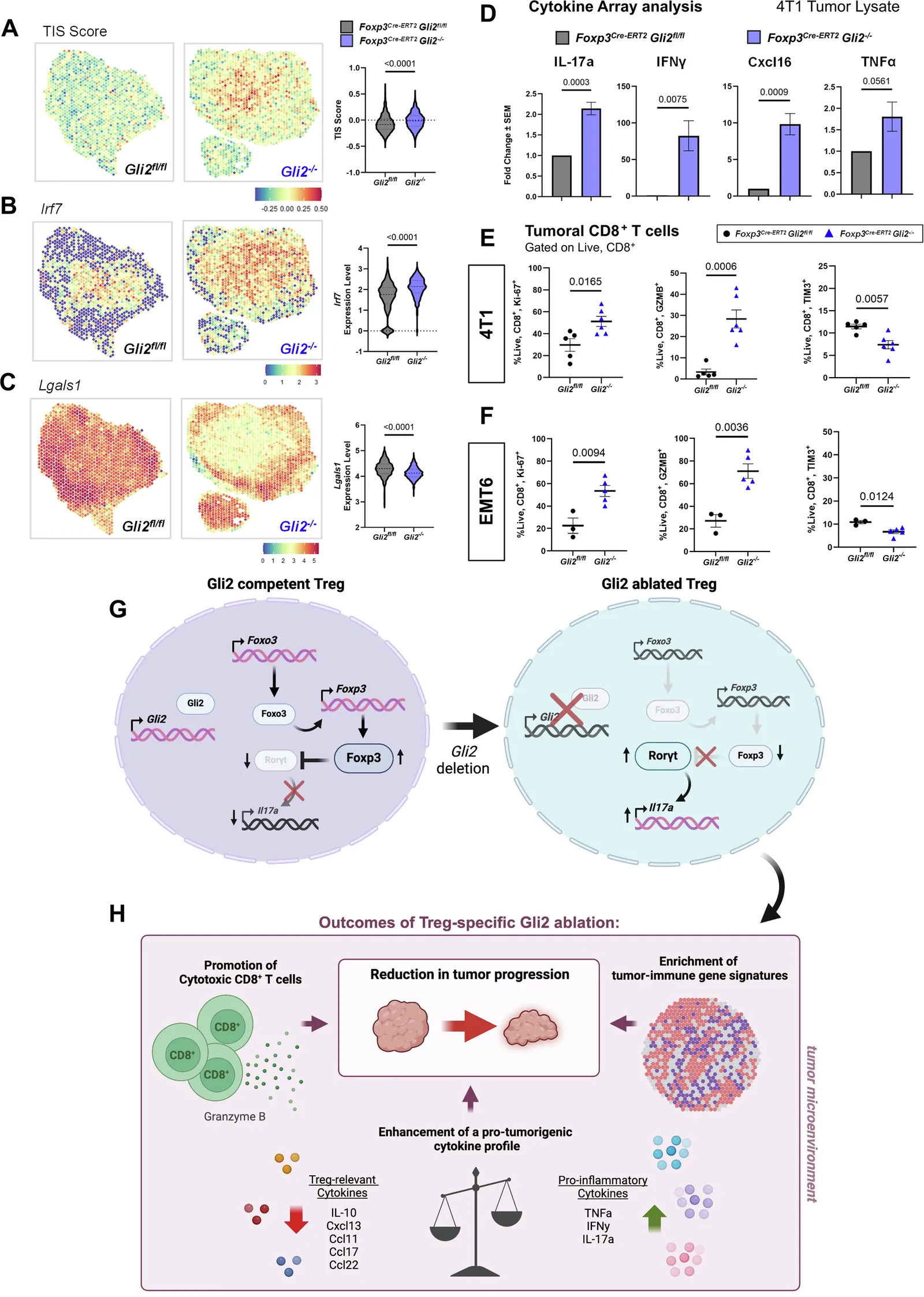
Gli2 ablation in Tregs promotes an inflammatory TME. Representative spatial dimensional plots of the **A** TIS score, **B** *Irf7*, and **C** *Lgals1* generated using the *AddModuleScore* function in Seurat v5. The score or normalized expression level of each barcode/spot parsed out by treatment was plotted for each feature (right). **D** Cytokine array data of inflammatory cytokines (IL-17a, IFNγ, Cxcl16, TNFα) in Foxp3-*Gli2*^*fl/fl*^ and Foxp3-*Gli2*^*−/−*^ tumor lysates. **E**, **F** Flow cytometric analyses were performed to query CD8^+^ T cell populations in the tumors of 4T1 or EMT6 Foxp3-*Gli2*^*−/−*^ and control mice. **E** Overall Ki-67^+^, Granzyme B (GZMB^+^), and TIM3^+^ CD8^+^ T cells in 4T1 and **F** EMT6 primary tumors. **G** Graphical summary of mechanism at which Gli2 modulates the activity of Foxo3 and Rorγt, influencing the transcriptional activity of *Foxp3* and *Il17a*, respectively. **H** Overview of the systemic changes in the TME as a result of the Gli2 ablation in Tregs. For 10X Visium analyses, *n* = 2 Foxp3-*Gli2*^*fl/fl*^, 2 Foxp3-*Gli2*^*−/−*^. Cytokine array data is representative of n = 3 Foxp3-*Gli2*^*fl/fl*^, 3 Foxp3-*Gli2*^*−/−*^. For 4T1 tumor studies, *n* = 5 Veh, 6 Foxp3-*Gli2*^*−/−*^. For EMT6 tumor studies, *n* = 3 Foxp3-*Gli2*^*fl/fl*^, 5 Foxp3-*Gli2*^*−/−*^. Data representative of two repeats.

GSEA was also performed in each zone to assess enriched GO terms in the Foxp3-*Gli2*^*−/−*^ tumors versus control, revealing that NF-κB signaling and other inflammatory-related processes are enriched in the “Tumor” zone of Foxp3-*Gli2*^*−/−*^ tumors (Supplementary Fig. 9G–J). A KEGG pathway analysis using the DEGs of the “Tumor” enriched zone between Foxp3-*Gli2*^*fl/fl*^ and Foxp3-*Gli2*^*−/−*^ tumors revealed that ‘Antigen processing and presentation’ and ‘Natural Killer cell-mediated cytotoxicity’ were significantly upregulated in the “Tumor” enriched zone of Foxp3-*Gli2*^*−/−*^ tumors (Supplementary Fig. 9K). These GSEA data suggest that Gli2 ablation in Tregs likely influences the immunogenicity of the tumor and promotes an inflammatory milieu of the TME.

### Gli2 ablation in Tregs triggers enhanced infiltration of cytotoxic CD8^+^ T cells into the tumor

To assess the immunogenicity of the tumor due to Foxp3-*Gli2*^*−/−*^ Tregs, we applied the tumor inflammatory signature (TIS) score, a pan-cancer metric associated with improved prognosis and immunotherapy in multiple cancers, including breast cancer. A high TIS score correlates with an inflammatory tumor milieu and increased sensitivity to pembrolizumab immunotherapy [61]. The TIS score was applied to each cluster in the dataset. This revealed that cluster 0, the “Tumor-enriched” zone, and cluster 3, a cluster within the “Tumor-Immune” enriched zone, hold the highest TIS scores as compared to other clusters (Supplementary Fig. 10A). Additionally, when comparing the overall score from all zones across all tumors, there was a significantly higher TIS score in the Foxp3-*Gli2*^*−/−*^ tumors, as compared to Foxp3-*Gli2*^*fl/fl*^ control tumors (Fig. 6A). When parsing out the TIS score by cluster and treatment, Foxp3-*Gli2*^*−/−*^ tumors had a significantly higher TIS score when comparing clusters 0, 5, and 6, which are associated with the “Tumor-enriched” and “Tumor-Macrophage-enriched” zones (Supplementary Fig. 10B). These TIS score data suggest that the impaired immunosuppression and enhanced inflammatory activity of Foxp3-*Gli2*^*−/−*^ Tregs may be sufficiently impactful in modifying the immune status of tumor.

To identify specific genes differentially expressed in the Foxp3-*Gli2*^*−/−*^ tumors that may be contributing to this transition in a more inflammatory TME, top DEGs were identified between the Foxp3-*Gli2*^*fl/fl*^ and Foxp3-*Gli2*^*−/−*^ tumors and genes associated with immunogenicity and tumor-immune response were queried. *Irf7*, *Lgals1*, and *Arg1* were identified as top DEGs associated with immunosuppression, interferon signaling, and tumor escape (Supplementary Table 3). Interferon regulatory factor 7 (Irf7) is essential for type I interferon signaling and has been highlighted to play different roles in tumorigenesis. In breast cancer, *Irf7* can inhibit bone metastasis through immune escape and by promoting NK cell-mediated cytotoxicity [62, 63]. In Foxp3-*Gli2*^−/−^ tumors, there was a significant increase in *Irf7* expression as compared to control and the intensity of its expression was most profound in the “Tumor” enriched zone (Fig. 6B). This evidence underscores the previous finding that this zone is enriched in GO biological processes involving NK-cell mediated cytotoxicity and interferon-related processes, especially in Foxp3-*Gli2*^*−/−*^ tumors (Supplementary Figs. 8E and 9I). *Lgals1* encodes for galactin-1, a glycan-binding protein associated with immunosuppression and metastasis in breast cancer. The expression of galactin-1 is positively correlated with the frequency of CD4^+^ CD25^+^ Foxp3^+^ Tregs in primary tumors, metastatic, and secondary lymphoid tissues in different cancer models, including 4T1 [64]. A reduction in *Lgals1* expression was registered in the Foxp3-*Gli2*^*−/−*^ tumors as compared to tumors from Foxp3-*Gli2*^*fl/fl*^ mice. The decreased expression of *Lgals1* was also highly localized to the “Tumor” enriched zone (Fig. 6C). *Arg1* encodes Arginase-1, an enzyme associated with the immunosuppressive M2 macrophage subset [65]. In the bordering “Immune” and “Tumor_Macrophage” enriched zones of the TME, zones where a macrophage signature was enriched, there was a reduction in *Arg1* expression in Foxp3-*Gli2*^*−/−*^ tumors (Supplementary Fig. 10C). Altogether, these changes in immunostimulatory and immunosuppressive-related signatures illustrate the significant alterations driven by *Gli2*^*−/−*^ *Tregs* in the TME.

Given that the inferences from the spatial transcriptomic analyzes are limited due to the small sample size and inter-tumor variability, it was essential to conduct more in-depth assessments to determine how this specific ablation in Tregs impacts other factors of the TME. We next assessed other factors of the TME to further identify the secondary effects of Gli2-ablated Tregs and how their impaired immunosuppressive activity induces a more inflammatory TME. In agreement with this, there was a significant increase in inflammatory cytokines associated with Th17 cells and other tumor-infiltrating lymphocytes such as CD8^+^ T cells (IL-17a, IFNγ, Cxcl16, and TNFα) in the 4T1 tumors from Foxp3-*Gli2*^*−/−*^ mice (Fig. 6D) [66–68]. Considering these findings and the data demonstrating a stark reduction in tumor growth in Foxp3-*Gli2*^*−/−*^ mice (Fig. 4B, and Supplementary Fig. 5A), we queried the frequency, activation, and exhaustion of cytotoxic CD8^+^ T cells in the primary tumor. While the overall frequency of CD8^+^ T cells was comparable, there was a significant increase in proliferative (Ki-67^+^) and cytotoxic (granzyme B^+^) CD8^+^ T cells, and a remarkable reduction in exhausted (TIM3^+^) CD8^+^ T cells in both 4T1 and EMT6 tumors from Foxp3-*Gli2*^*−/−*^ mice as compared to Foxp3-*Gli2*^*fl/fl*^ mice (Fig. 6E, F, and Supplementary Fig. 10D). In summary, Treg-specific deletion of Gli2 spurs trans-differentiation of Tregs to Tr17/Th17 cells (Fig. 6G) inciting remarkable changes in the mammary TME including inflammatory cytokines and elevated abundance of cytotoxic CD8^+^ T cells (Fig. 6H). The biological variations noted between Foxp3-*Gli2*^*−/−*^ and control tumors in the spatial transcriptomics align with these pro-inflammatory changes in the TME. Altogether, this work suggests that Gli2 signaling plays a significant role in modulating immunosuppression in the TME via Tregs, contributing to the immunosuppressive, immunologically cold milieu.

## Discussion

Using a novel Foxp3-*Gli2*^*fl/fl*^ mouse model, our work revealed that ablation of Gli2 in Tregs induced a pro-inflammatory, Th17-like phenotype in Tregs (Tr17), evidenced by an increase in Rorγt and IL-17a expression. These studies revealed a dual effect of Gli2 ablation in transcriptionally restricting Foxp3 and while relieving repression of Rorγt expression, thereby prompting a pro-inflammatory, Th17-like phenotype in Tregs. Remarkably, the effect of Gli2 ablation in Tregs was far-reaching and not limited to Tregs. Gli2 ablation of Tregs transformed the tumor milieu, highlighted by the enrichment of inflammatory cytokines such as IFNγ, CXCL16, and TNFα, which promote the recruitment and activity of inflammatory Th17s and CD8^+^ T cells, simultaneously with a decrease in cytokines such as CCL11, CCL22, and IL-10, which govern Treg activity and retention in the TME.

The role of Hh signaling in T cell biology has been investigated in the context of T cell development, inflammatory activity, immunosuppression, and plasticity. Hh signaling is essential in the thymic development of CD4^+^ T cells and the generation and maintenance of helper CD4^+^ T cell subsets such as Th1, Th2, Th17, and Tregs [20, 25, 26, 32, 69, 70]. Foxp3 is the nidus of the identity of Tregs. Foxp3 is highly critical for the generation of CD4^+^ CD25^+^ Tregs, and its impairment causes dysfunction in the suppressor program in Tregs. In fact, ectopic expression of Foxp3 can induce immunosuppressive activity in CD4^+^ CD25^-^ Tregs [15]. In Treg biology, the durability of Foxp3 expression or its loss regulates their plasticity. For example, co-culture of Tregs with inflammatory cytokines, IL-1 and IL-6, reduces Foxp3 expression and promotes the production of IL-17 [18]. Foxp3 also solidifies Tregs by antagonizing the expression of Th17-specific transcription factors Rorγt and Rorα [18]. Thus, molecular switches and environmental cues are highly involved in governing the plasticity and function of Tregs. Deleting Gli2 decreased Foxp3 and reduced the expression of classical Treg phenotypic markers Ebi3, TGF-β, CD25, and IL-10, which are directly regulated by Foxp3, and studies have shown that the disturbance of Foxp3 activity can precipitate instability in Treg phenotype and function [15].

Tregs have distinct metabolic vulnerabilities that may affect their immunosuppressive function. They rely upon glycolysis for proliferation and lipid oxidation for their suppressive activity [54–58, 71–73]. Gli2 ablation promoted enrichment of oxidative phosphorylation and diminished a FAO profile in Tregs, highlighting that Gli2 is a critical regulator of Treg stability and suppressive functionality, with its loss executing their plasticity to evolve into Tr17/Th17 cells [57, 71]. Th17 and Tr17 cells produce inflammatory cytokines, including IL-17, IFNγ, and GM-CSF, all of which can have diverse effects on the TME depending on the pathology. The ability for tumoral Treg cells to adapt Tr17 attributes anchors Th17-driven immune function and disease processes [12].

In colorectal carcinoma, non-small cell lung cancer, and lymphomas, Th17 cells exhibit pathogenic functions as IL-17a is known to promote tumor growth, angiogenesis, and metastasis in the absence of infiltrating lymphocytes [69, 73–75]. In TNBC, the infiltration of inflammatory cytokine-producing Th17 cells in the tumor is accompanied by a favorable prognosis as their presence and activity promote an anti-tumor milieu by stimulating antigen-specific cytotoxic CD8^+^ T cells [73–76]. Additionally, a Th17 metagene analysis has been shown to be a novel prognostic composite biomarker in T cell non-inflamed TNBC. The presence of an enriched Th17 signature was associated with a highly favorable prognosis in TNBC tumors with low T cell infiltration [73].

The regulation of Th17 activity through Hh signaling is dependent on the pathological context. In irritable bowel disease, ablation of Ihh and Smo impaired Th17 cells and promoted Tregs [69, 70]. In atopic dermatitis, Gli2 signaling is essential in activating skin Tregs, where the repression of Gli2 led to exacerbated skin inflammation [32]. In the context of intestinal inflammation, Vismodegib treatment reduced pathogenic Th17 cells, but in the context of mammary cancer, Vismodegib increased pro-inflammatory Th17s [22, 69]. Spatial transcriptomics enabled the mapping of unique transcriptome regions marked by immune infiltration and revealed the positive enrichment of signatures involved in immune response, antigen presentation, and interferon signaling in tumor-rich regions of the TME in the context of Gli2 ablation in Tregs. Known regulators of immunosuppression, such as Gal-1 (*Lgals1*) and Arg-1, were notably reduced in Foxp3-*Gli2*^*−/−*^ tumors. In conjunction, the enrichment of inflammatory signatures such as the TIS score and the increased infiltration of cytotoxic CD8^+^ T cells in Foxp3-*Gli2*^*−/−*^ tumors also highlight the immune status shift induced by Gli2 ablation in Tregs.

As such, these data underscore the critical role of Gli2 in governing the status, stability, and functionality of Tregs. Given the central role of Tregs in providing self-tolerance and supporting robust immunosuppression, the findings present Gli2 and, by extension, Hh activity as a key determinant of Treg immunosuppressive function. Beyond tumor biology, these findings hold important implications in our understanding of Tregs in maintaining a tolerogenic environment during pregnancy or during allogenic transplantation or in controlling autoreactivity for treating autoimmune disorders.

In the tumor milieu, the repercussions of this single modification in Tregs are impactful and induce major restructuring of the cellular and acellular constituents that relieve the immunologically cold tumor environment. These findings support the possibility of considering Hh signaling as a potential target in T cell-based therapy, especially in priming the TME to be more responsive and receptive to potential adjuvant treatments.

## Supplementary information

Original Data

Supplemental Figure 1

Supplemental Figure 2

Supplemental Figure 3

Supplemental Figure 4

Supplemental Figure 5

Supplemental Figure 6

Supplemental Figure 7

Supplemental Figure 8

Supplemental Figure 9

Supplemental Figure 10

Supplemental Table 1

Supplemental Table 2

Supplemental Table 3

Supplemental Table 4

Supplemental Table 5

Original Data_All PCR Raw Data Files_GEMM manuscript

## Data Availability

The bulk RNA-Seq, spatial transcriptomics, and R code generated from this work are available from the corresponding author upon reasonable request.
