# Supplementary material for: Disrupting Treg lineage stability elicits RORgT-mediated plasticity and enhances anti-tumor immunity
Source: Cell Death Dis. 2026 Jul 6;17(1):836. doi: 10.1038/s41419-026-09025-0 (PMC13616968; doi:10.1038/s41419-026-09025-0)

All Original Images

Figure 1D

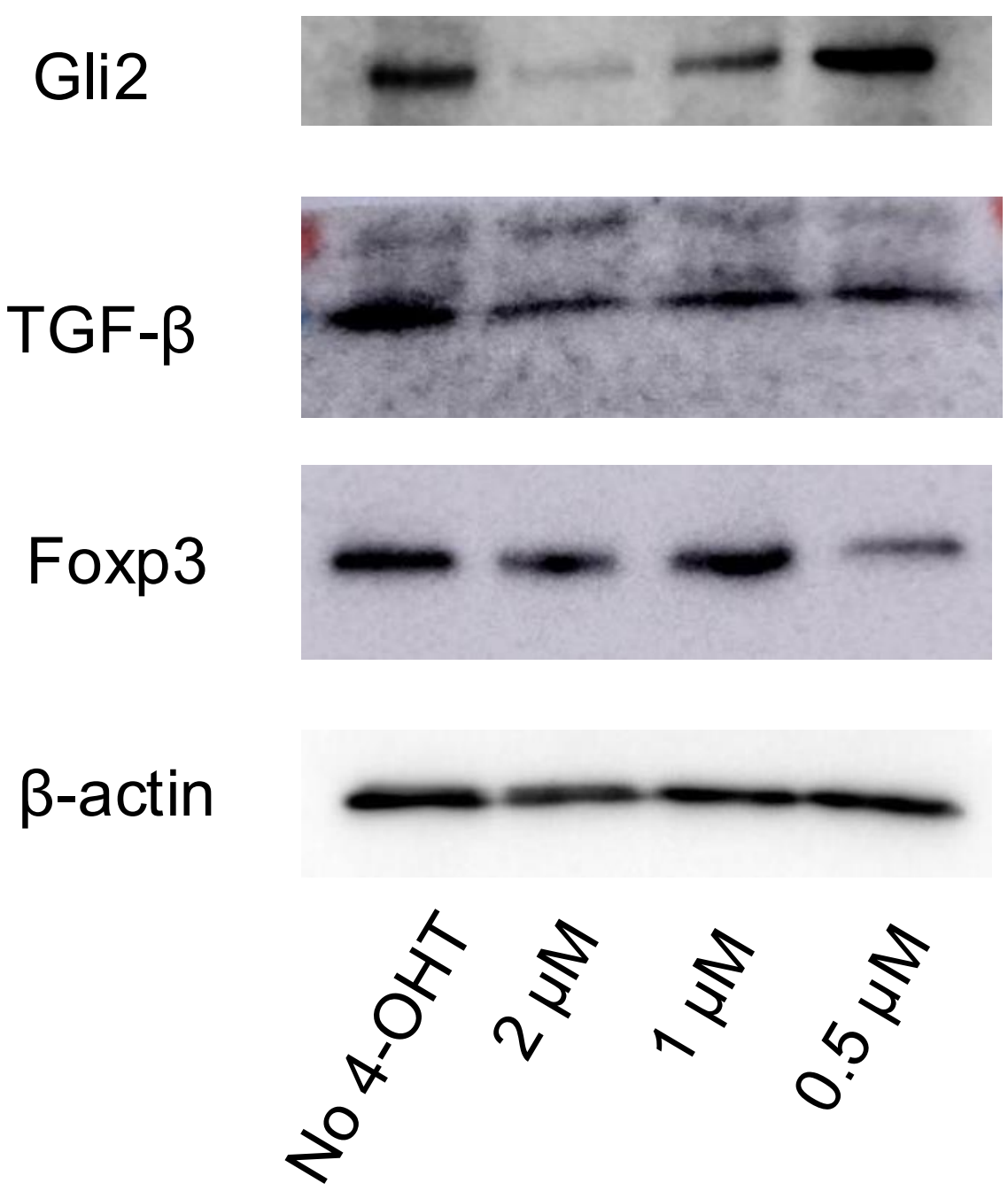

Figure 1G

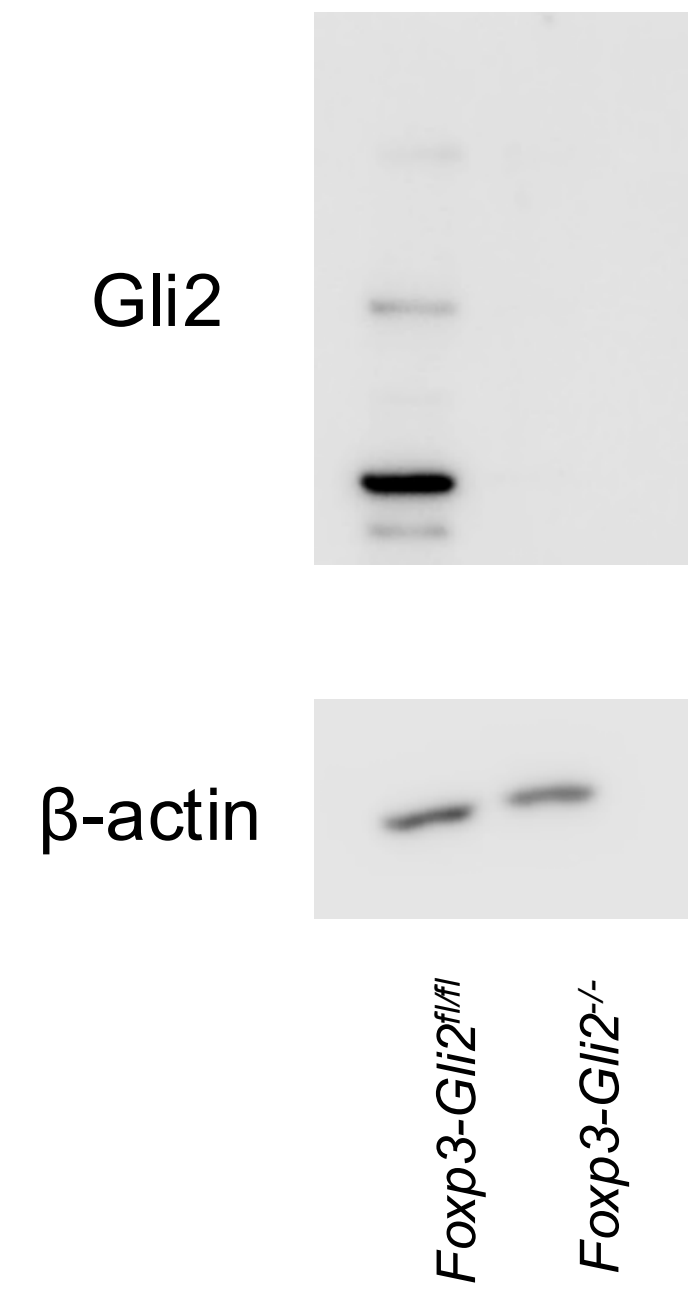

Supplemental Figure 1C

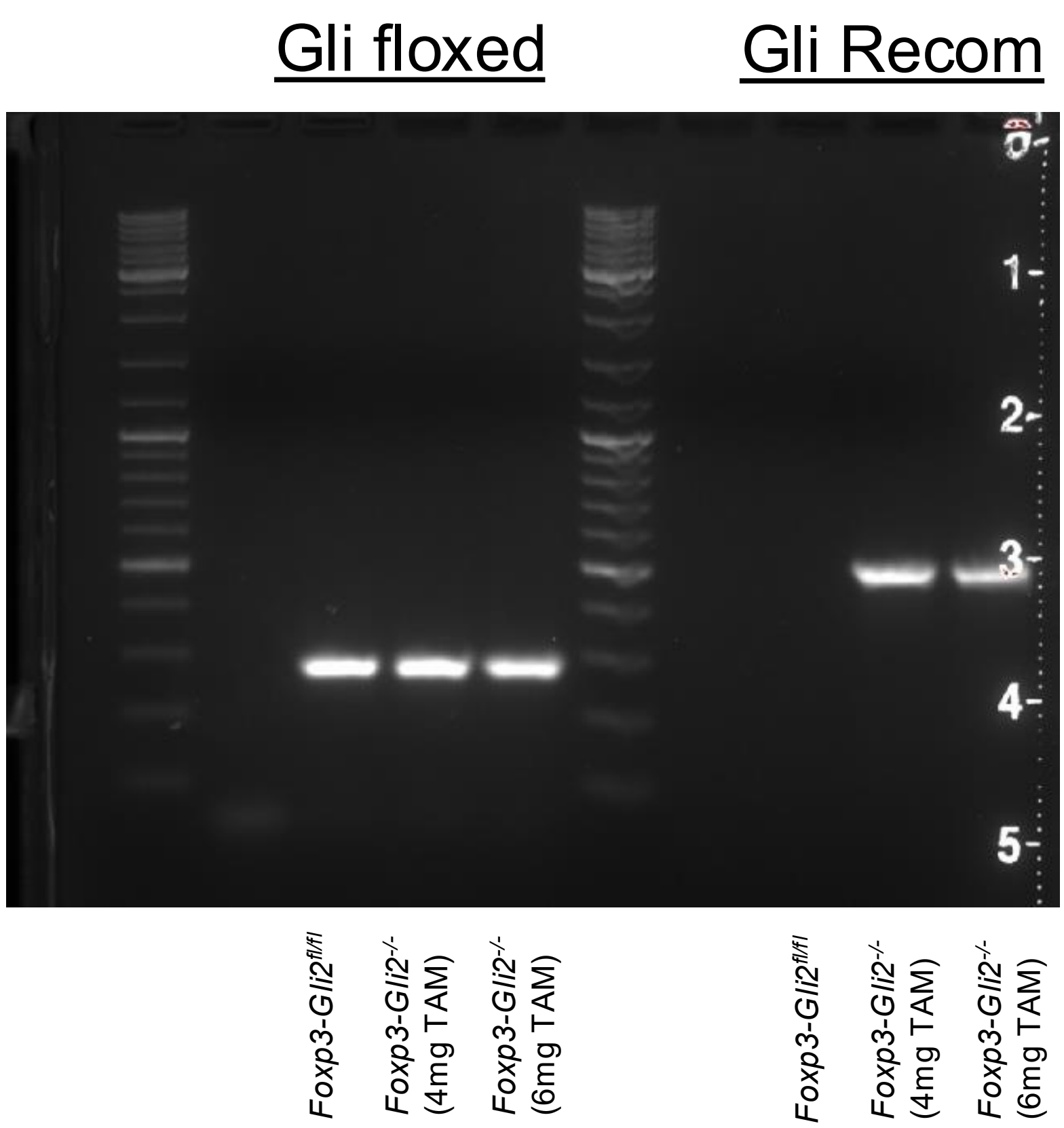

Supplement: Supplementary file 3 [file 41419_2026_9025_MOESM1_ESM.pdf]
