# Supplementary material for: Disrupting Treg lineage stability elicits RORgT-mediated plasticity and enhances anti-tumor immunity
Source: Cell Death Dis. 2026 Jul 6;17(1):836. doi: 10.1038/s41419-026-09025-0 (PMC13616968; doi:10.1038/s41419-026-09025-0)

Supplemental Figure 8

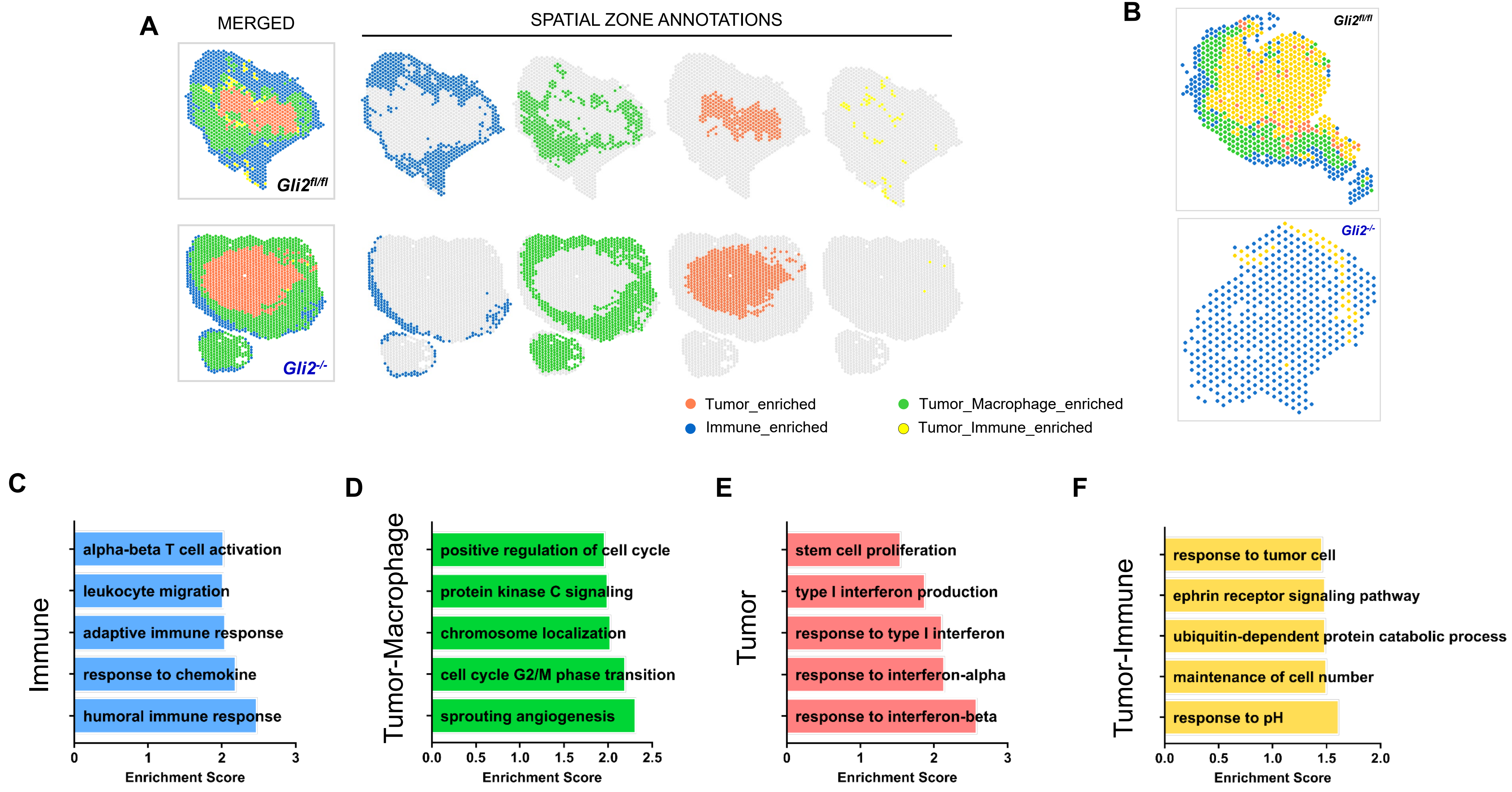

Supplement: Supplementary file 11 [file 41419_2026_9025_MOESM9_ESM.pdf]
