# Supplementary material for: Disrupting Treg lineage stability elicits RORgT-mediated plasticity and enhances anti-tumor immunity
Source: Cell Death Dis. 2026 Jul 6;17(1):836. doi: 10.1038/s41419-026-09025-0 (PMC13616968; doi:10.1038/s41419-026-09025-0)
Supplement: Supplementary file 12 [file 41419_2026_9025_MOESM12_ESM.docx]

**Supplementary Table 1**. Reagents and Tools

| **REAGENT or RESOURCE** | **SOURCE** | **IDENTIFIER** |
| --- | --- | --- |
| Antibodies | | |
| Anti-Foxp3 Rabbit mAb | Abcam, Cambridge, MA | Cat#ab215206 |
| Anti-TGFβ Rabbit mAb | Cell Signaling Technologies, Danvers, MA | Cat#5544S |
| ROR gamma (t) Monoclonal Antibody (AFKJS-9), eBioscience™, Invitrogen™ | Fisher Scientific | Cat #50-135-65 |
| Rat IgG2a kappa Isotype Control (eBR2a), eBioscience™, Invitrogen™ | Fisher Scientific | Cat#501129472 |
| Anti-mouse CD8a-FITC  Clone 53-6.7 | Biolegend, San Diego, CA | Cat#100706 |
| Anti-mouse CD8a-PE/Cy7  Clone 53-6.7 | Biolegend, San Diego, CA | Cat#100722 |
| Anti-mouse CD4-APC/Cy7  Clone GK1.5 | Biolegend, San Diego, CA | Cat#100414 |
| Anti-mouse Foxp3-APC  Clone FJK-16s | Fisher Scientific, Hampton, NH | Cat#17577382 |
| Anti-mouse Granzyme B-PE  Clone QA16A02 | Biolegend, San Diego, CA | Cat#372208 |
| Anti-mouse Rorγt-PE  Clone AFKJS-9 | Fisher Scientific, Hampton, NH | Cat#12698882 |
| Anti-mouse IFNγ-BV510  Clone XMG1.2 | Biolegend, San Diego, CA | Cat#505842 |
| Anti-mouse IL-17a-BV711  Clone TC11-18H10.1 | Biolegend, San Diego, CA | Cat#506941 |
| Anti-mouse Ki-67-BV605  Clone 16A8 | Biolegend, San Diego, CA | Cat#652413 |
| Anti-mouse CD45.2- FITC  Clone 104 | Biolegend, San Diego, CA | Cat#109806 |
| Purified anti-mouse CD3 Antibody  Clone 17A2 | Biolegend, San Diego, CA | Cat#100202 |
| Purified anti-mouse CD28 Antibody  Clone 37.51 | Biolegend, San Diego, CA | Cat#102102 |
| Purified anti-mouse IL-4 Antibody  Clone 11B11 | Biolegend, San Diego, CA | Cat#504135 |
| Purified anti-mouse IFNγ Antibody  Clone R4-6A2 | Biolegend, San Diego, CA | Cat#505710 |
| **Biological Samples** |  |  |
| Splenic Naïve CD4^+^ T cells and Tregs | 6-8 week-old C57/BL6J Mice from Jackson Labs | Stock#000664 |
| **Reagents & Pharmacologic Agents** | | |
| Recombinant Mouse IL-2 (rmIL-2) | R&D Systems, Minneapolis, MN | Cat#402ML100 |
| Recombinant human TGFβ1 (rhTGFβ1) | R&D Systems, Minneapolis, MN | Cat#240B010 |
| GANT61 GLI inhibitor | Tocris Bioscience, Bristol, United Kingdom | Cat#319110 |
| Vismodegib Smoothened Inhibitor | Selleck Chemicals, Houston, TX | Cat#S1082 |
| Cell Activation Cocktail (with Brefeldin A) | Biolegend, San Diego, CA | Cat#423304 |
| InSolution™ Tamoxifen, 4-Hydroxy-, (Z) (4-OHT) | Sigma-Aldrich, St. Louis, MO | Cat #508225 |
| Tamoxifen | Sigma-Aldrich, St. Louis, MO | Cat#T5648 |
| ACK red blood cell lysis buffer | Lonza, Basel, Switzerland | Cat#10-548E |
| **Critical Commercial Assays** | | |
| RNeasy Mini Kit for RNA extraction | Qiagen, Valencia, CA | Cat#74106 |
| High Capacity cDNA Reverse Transcription Kit for cDNA synthesis | Life Technologies, Carlsbad, CA | Cat#4374967 |
| CFSE Cell Division Tracker Kit | Biolegend, San Diego, CA | Cat#423801 |
| Proteome Profiler Mouse XL Cytokine Array | R&D Systems, Minneapolis, MN | Cat#ARY028 |
| True Nuclear Transcription factor buffer set | Biolegend, San Diego, CA | Cat#424401 |
| Naïve CD4+ T Cell Isolation Kit, Mouse | Miltenyi Biotec, San Diego, CA | Cat#130104453 |
| Tumor Dissociation Kit, mouse | Miltenyi Biotec, San Diego, CA | Cat#130096730 |
| EasySep Mouse CD4+CD25+ Regulatory T cell Isolation Kit II | StemCell Technologies, Vancouver, Canada | Cat#18783 |
| **Deposited Data**  All datasets generated in this manuscript are available upon request.  Publicly available deposited data we utilized can be found:  RNA-seq data from basal cell carcinoma patients treated with Vismodegib: GSE58377  CHIP-Seq data of human THP-1 cells: GSM2212253 | | |
| **Experimental Models: Cell Lines** | | |
| Luciferase-expressing 4T1 Murine Mammary Carcinoma Cell Line | ATCC, Manassas, VA |  |
| Luciferase-expressing EMT6 Murine Mammary Carcinoma Cell Line | Sophia Ran, Ph.D., Carbondale, IL |  |
| EL4 Murine T Lymphoblast Lymphoma Cell Line | ATCC, Manassas, VA |  |
| **Experimental Models: Organisms/Strains** | | |
| Balb/c Mice | Jackson Labs, Bar Harbor, ME | Stock #000651 |
| C57/BL6J Mice | Jackson Labs, Bar Harbor, ME | Stock #000664 |
| Gli2^flox^ mutant mice | Alexandra L. Joyner, Memorial Sloan-Kettering Cancer Center, New York, NY |  |
| **Other** |  |  |
| ACK red blood cell lysis buffer | Lonza, Basel, Switzerland | Cat#10-548E |
| **Primers** | | |
| Mouse *Tgfb1* | Life Technologies, Carlsbad, CA | Mm01178820_m1 |
| Mouse *Rorc* | Life Technologies, Carlsbad, CA | Mm01261022_m1 |
| Mouse *Ebi3* | Life Technologies, Carlsbad, CA | Mm00469294_m1 |
| Mouse *Il2ra* | Life Technologies, Carlsbad, CA | Mm01340213_m1 |
| Mouse *Foxo1* | Life Technologies, Carlsbad, CA | Mm00490671_m1 |
| Mouse *Foxo3* | Life Technologies, Carlsbad, CA | Mm01185722_m1 |
| Mouse *Gli1* | Life Technologies, Carlsbad, CA | Mm00494654_m1 |
| Mouse *Gli2* Synthesized Primers | Eurofins Genomics, Louisville, KY | *Forward*: GGGACTCTTTAGCCTCGCAG  *Reverse*: ATGACTACCTCAACCCTGTGG |
| Mouse *Il17a* | Life Technologies, Carlsbad, CA | Mm00439618_m1 |
| Mouse *Actb* | Life Technologies, Carlsbad, CA | Mm02619580_m1 |
| **Software** | | |
| GraphPad Prism 10 Software | GraphPad Software, Inc., Boston, MA | https://www.graphpad.com |
| FlowJo 10 Software | BD Biosciences, San Jose, CA | https://www.flowjo.com/solutions/flowjo |
| RStudio v4.3.1 | Posit PBC, Boston, MA | https://posit.co/ |
| Loupe Browser v8 | 10x Genomics, Pleasanton, CA | https://10xgenomics.com/ |
| Gene Set Enrichment Analysis (GSEA) Software | Broad Institute and University of California San Diego, San Diego, CA | https://www.gsea-msigdb.org/gsea/ |
