# Supplementary material for: Disrupting Treg lineage stability elicits RORgT-mediated plasticity and enhances anti-tumor immunity
Source: Cell Death Dis. 2026 Jul 6;17(1):836. doi: 10.1038/s41419-026-09025-0 (PMC13616968; doi:10.1038/s41419-026-09025-0)
Supplement: Supplementary file 13 [file 41419_2026_9025_MOESM16_ESM.docx]

**Supplemental Table 5. Primer Sequences for ChIP-qPCR**

Primer pairs to detect Foxo3 occupancy on the *Foxp3* promoter used were as follows:

|  | **Forward** | **Reverse** |
| --- | --- | --- |
| **CNS1**  **(or Promoter Binding Site)** | ACCCTGCAATTATCAGCACAC | GAAGTGGACTGCTAGAGGGG |
| **CNS3**  **(or Intron 1 Binding Site)** | GTTGCCGATGAAGCCCAATG | TTCCTGCACTGTCTGTTGGG |

Primer pairs to detect Rorγt occupancy on the *Il17a* promoter used were as follows:

|  | **Forward** | **Reverse** |
| --- | --- | --- |
| **CNS2** | ATGGGCCTCTCTTTCCACTGATG | GGAATTTGTGGTGGAAGGGAGTG |
| **BS2** | GCAAAGCATCTCTGTTCAGCT | CTTACGTCAAGAGTGGGTTGG |
| **BS3** | GGTACATGTTTGACTGTGCACG | GTCTTAGACAGCAGTGGTCCT |

**Note: CNS, conserved non-coding sequence; BS, binding site**
